# Paraspeckle Protein NONO Promotes TAZ Phase Separation in the Nucleus to Drive the Oncogenic Transcriptional Program

**DOI:** 10.1002/advs.202102653

**Published:** 2021-10-29

**Authors:** Yiju Wei, Huacheng Luo, Patricia P. Yee, Lijun Zhang, Zhijun Liu, Haiyan Zheng, Lei Zhang, Benjamin Anderson, Miaolu Tang, Suming Huang, Wei Li

**Affiliations:** ^1^ Division of Pediatric Hematology and Oncology Department of Pediatrics Penn State Health Hershey Medical Center Penn State College of Medicine Hershey PA 17033 USA; ^2^ Department of Pharmacology Penn State Health Hershey Medical Center Penn State College of Medicine Hershey PA 17033 USA; ^3^ Institute for Personalized Medicine Penn State Health Hershey Medical Center Penn State College of Medicine Hershey PA 17033 USA; ^4^ Department of Biochemistry & Molecular Biology Penn State Health Hershey Medical Center Penn State College of Medicine Hershey PA 17033 USA; ^5^ Biological Mass Spectrometry Facility Robert Wood Johnson Medical School, Rutgers The State University of New Jersey Piscataway NJ 08854 USA; ^6^ Hepatic Surgery Center Tongji Hospital Tongji Medical College Huazhong University of Science and Technology Wuhan Hubei Province 430030 China; ^7^ Penn State Cancer Institute Penn State Health Hershey Medical Center Penn State College of Medicine Hershey PA 17033 USA

**Keywords:** glioblastoma, Hippo pathway, liquid–liquid phase separation, NONO, TAZ, transcription regulation

## Abstract

The Hippo pathway effector TAZ promotes cellular growth, survival, and stemness through regulating gene transcription. Recent studies suggest that TAZ liquid–liquid phase separation (LLPS) compartmentalizes key cofactors to activate transcription. However, how TAZ LLPS is achieved remains unknown. Here, it is shown that the paraspeckle protein NONO is required for TAZ LLPS and activation in the nucleus. NONO is a TAZ‐binding protein. Their interaction shows temporal regulation parallel to the interaction between TAZ and TEAD as well as to the expression of TAZ target genes. NONO depletion reduces nuclear TAZ LLPS, while ectopic NONO expression promotes the LLPS. Accordingly, NONO depletion reduces TAZ interactions with TEAD, Rpb1, and enhancers. In glioblastoma, expressions of NONO and TAZ are both upregulated and predict poor prognosis. Silencing NONO expression in an orthotopic glioblastoma mouse model inhibits TAZ‐driven tumorigenesis. Together, this study suggests that NONO is a nuclear factor that promotes TAZ LLPS and TAZ‐driven oncogenic transcriptional program.

## Graphical abstract

The Hippo pathway effector TAZ activates transcription by compartmentalizing key cofactors through liquid–liquid phase separation (LLPS). How TAZ LLPS is achieved remains unknown. This study shows that the nuclear paraspeckle protein NONO, a TAZ‐binding protein, is required for TAZ LLPS and activation in the nucleus, as well as TAZ‐driven oncogenic transcriptional program in glioblastoma tumorigenesis.

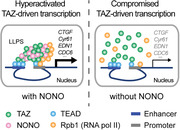

## 1 Introduction

Transcriptional coactivator with PDZ‐binding motif (TAZ) and its paralogue, yes‐associated protein (YAP), are two nuclear effectors of the Hippo pathway, a conserved signaling network regulating cellular growth, survival, and stemness.^[^ ^1^ ^]^ In this pathway, TAZ and YAP are controlled by a serine/threonine kinase cascade composed of MST1/2 kinases and their substrates, Lats1/2 kinases. Phosphorylation of TAZ and YAP by Lats1/2 results in their cytoplasmic retention and degradation through the ubiquitin‐proteasome‐mediated mechanism. Dephosphorylated TAZ and YAP enter the nucleus to access their target genes largely through binding to the TEAD family of transcription factors. TAZ and YAP can activate transcription through recruiting several transcriptional cofactors to enhancer regions.^[^ ^2^, ^3^, ^4^, ^5^ ^]^ Recent studies revealed that TAZ and YAP can form LLPS condensates in the nucleus.^[^ ^6^, ^7^ ^]^ The nuclear TAZ or YAP condensates are enriched by enhancers and several transcriptional regulators, such as TEAD, BRD4, MED1, CDK9, and RNAP II.^[^ ^6^, ^7^ ^]^ It has been proposed that LLPS of TAZ or YAP is involved in compartmentalizing these key transcription cofactors and activating transcription. Although these studies have found that several factors, such as osmotic stress, protein and salt concentrations, as well as the Hippo signaling, can affect TAZ or YAP LLPS, it is still unknown if any nuclear factors could regulate their LLPS.

Glioblastoma (GBM) is the most lethal and most common primary brain cancer in adults. Current treatment includes surgery followed by a combination of chemotherapy and adjuvant radiotherapy. In spite of these measures, median survival of GBM patients is approximately 18 months.^[^ ^8^ ^]^ Therapies targeting the underlying oncogenic signaling machinery may improve the prognosis of certain types of GBM. Molecular pathology studies classified GBM into subtypes differing in treatment responses and survival rates.^[^ ^9^, ^10^ ^]^ Among these, the mesenchymal (MES) subtype is the most aggressive. Previous studies have identified several transcriptional regulators, including CCAAT‐enhancer‐binding protein beta (C/EBP‐beta), signal transducer and activator of transcription 3 (STAT3), and TAZ, that drive GBM MES differentiation.^[^ ^11^, ^12^ ^]^ Therefore, inhibition of these aforementioned regulators may reduce the aggressiveness of MES GBM. It is still unclear if a distortion of the canonical Hippo pathway, contributing to aberrant TAZ activation, exists in GBM. However, previous studies showed that CpG island hypermethylation of the TAZ promoter exists in proneural (PN) but not MES GBM.^[^ ^11^ ^]^ This is consistent with increased expression of TAZ in MES GBM and enhanced activity of the TAZ‐TEAD transcriptional machinery.^[^ ^11^, ^13^ ^]^ Nonetheless, whether any other mediators are involved in the transcriptional regulatory machinery for MES GBM remains elusive.

Non‐POU domain‐containing octamer‐binding protein (NONO) is a nuclear paraspeckle protein belonging to the *Drosophila* behavior/human splicing (DBHS) protein family. Besides NONO, this family contains two other members, paraspeckle component 1 (PSPC1) and splicing factor proline/glutamine‐rich (SFPQ, also known as PSF). The DBHS proteins contain two RNA recognition motifs and are involved in transcriptional regulation, RNA processing, and DNA repair.^[^ ^14^ ^]^ NONO is ubiquitously expressed in most tissues,^[^ ^15^ ^]^ but its expression has been shown to increase in melanoma,^[^ ^16^ ^]^ breast cancers,^[^ ^17^ ^]^ and neuroblastoma.^[^ ^18^ ^]^ In these cancers, increased NONO expression correlates with tumor progression and aggressiveness. Still, whether NONO is upregulated and plays a role in GBM remains unknown.

Here, we used a proximity‐dependent biotinylation approach to search for TAZ‐binding proteins in GBM cells and identified NONO as a TAZ‐binding protein in the nucleus. Our results indicated that the presence of NONO is essential for TAZ to form LLPS condensates in the nucleus. In addition, we found that interactions between TAZ and TEAD as well as RNA polymerase II subunit B1 (Rpb1) are also enhanced by the presence of NONO. Our results suggest that NONO is a nuclear factor responsible for promoting TAZ LLPS and activation, thereby driving the GBM oncogenic transcriptional program.

## 2 Results

### 2.1 NONO is a TAZ‐Binding Protein in the Nucleus

**Figure 1:**
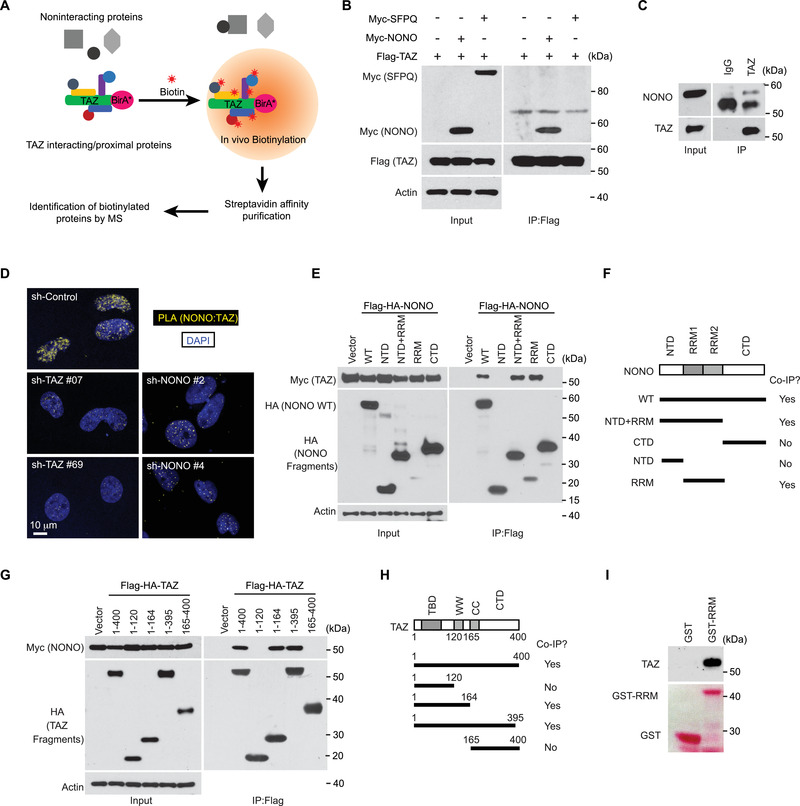
NONO is a TAZ‐binding protein in the nucleus. A) A diagram illustrating the usage of BioID to identify TAZ‐binding proteins. B) HEK293T cells were transfected with indicated genes. The cells were lysed and subjected to immunoprecipitation by a Flag antibody. The immunoprecipitated products were subjected to western blotting. C) LN229 cells were lysed and subjected to immunoprecipitation by aTAZ antibody or IgG control. The immunoprecipitated products were subjected to western blotting. D) LN229 cells stably transduced either with indicated shRNAs targeting TAZ or NONO or with a scrambled shRNA control and then subjected to PLA using TAZ and NONO antibodies. E) HEK293T cells were transfected with Myc‐tagged TAZ and Flag‐HA‐tagged NONO truncation mutants as indicated. The cells were lysed and subjected to immunoprecipitation by a Flag antibody. The immunoprecipitated products were subjected to western blotting. F) NONO truncational mutations and the co‐immunoprecipitation results observed in (E). G) HEK293T cells were transfected with Myc‐tagged NONO and Flag‐HA‐tagged TAZ truncational mutants as indicated. The cells were lysed and subjected to immunoprecipitation by a Flag antibody. The immunoprecipitated products were subjected to western blotting. H) TAZ truncation mutations and the co‐immunoprecipitation results observed in (G). I) Cell lysate from HEK293T cells was subjected to the GST pull down assay by purified GST or a fusion protein containing GST and the RRM domain of NONO (GST‐RRM). The precipitated products were subjected to western blotting. The upper panel was blotted by a TAZ antibody, and the lower one was stained by Ponceau S.

To understand how TAZ is regulated in the nucleus, we searched for proteins interacting with TAZ by a proximity‐dependent biotinylation approach known as BioID (**Figure** **1**A).^[^ ^19^ ^]^ TAZ was fused to BirA^R118G^, a mutant of the *E. coli* biotin ligase, and stably expressed in LN229 human glioblastoma cells (Figure S1A, Supporting Information). As controls, BirA^R118G^ or the empty vector was stably transduced into LN229 cells. After biotin‐streptavidin‐mediated purification, proteins were identified through mass spectrometry. Proteins more specifically enriched from the BirA^R118G^‐TAZ‐transduced cells were potential TAZ‐interacting proteins (Supplementary table 1). Some of them, e.g., AMOTL1, TEAD1, Arid1B, Arid1A, and ASPP2, are known to be TAZ‐binding proteins.^[^ ^20^, ^21^, ^22^, ^23^ ^]^ This observation indicated that the BioID system works appropriately. In addition to these proteins, NONO and SFPQ were among the enriched proteins (Supplementary table 1). Because NONO and SFPQ were previously shown to possess transcriptional regulatory functions, we were curious as to whether they could regulate TAZ.

Following BioID, we used immunoprecipitation to confirm the interaction between TAZ and NONO as well as between TAZ and SFPQ. Interestingly, NONO, but not SFPQ, was co‐immunoprecipitated by recombinant TAZ in HEK293T cells (Figure 1B). We also examined the possible interaction between TAZ and PSPC1, and found that TAZ interacts with NONO but not PSPC1 (Figure S1B, Supporting Information). These observations suggested that TAZ preferentially interacts with NONO rather than with the other two DBHS family members. Since YAP is a TAZ paralogue in the Hippo pathway, we examined whether NONO can also interact with YAP. Interestingly, YAP was also co‐immunoprecipitated by recombinant NONO, albeit stoichiometrically less than TAZ in HEK293T cells (Figure S1C, Supporting Information). This observation suggested that the interaction between YAP and NONO is weaker than that between TAZ and NONO. The interaction between TAZ and NONO was also detected at endogenous levels through co‐immunoprecipitation assays in LN229 cells (Figure 1C), suggesting that NONO and TAZ can interact with each other at physiological levels. To further examine this notion, we conducted the proximity ligation assay (PLA).^[^ ^24^ ^]^ PLA signal was observed in the nuclei of LN229 cells (Figure 1D; Figure S1D, Supporting Information). When NONO was knocked down by either of two different shRNAs or knocked out by CRISPR, the PLA signal was markedly reduced (Figure 1D; Figure S1D–F, Supporting Information). Likewise, when TAZ expression was silenced by either of two different shRNAs, the PLA signal was also eliminated (Figure 1D). These results indicated the PLA signal is specific for the NONO‐TAZ interaction, which occurs in the nucleus. To further characterize the TAZ‐NONO interaction, we mapped the domains in each protein responsible for their interaction. It appeared that the RRM domain in NONO (Figure 1E,F) and the WW domain in TAZ (Figure 1G,H) are responsible for the NONO‐TAZ interaction. We then conducted the GST pull down assay and found that GST‐RRM expressed from bacteria is able to pull down TAZ from HEK293T cell lysates (Figure 1I), suggesting that TAZ and NONO can directly bind to each other. Overall, these results identified that NONO is a TAZ‐binding protein in the nucleus.

### 2.2 NONO Interaction with TAZ is Associated with TAZ Activation

**Figure 2:**
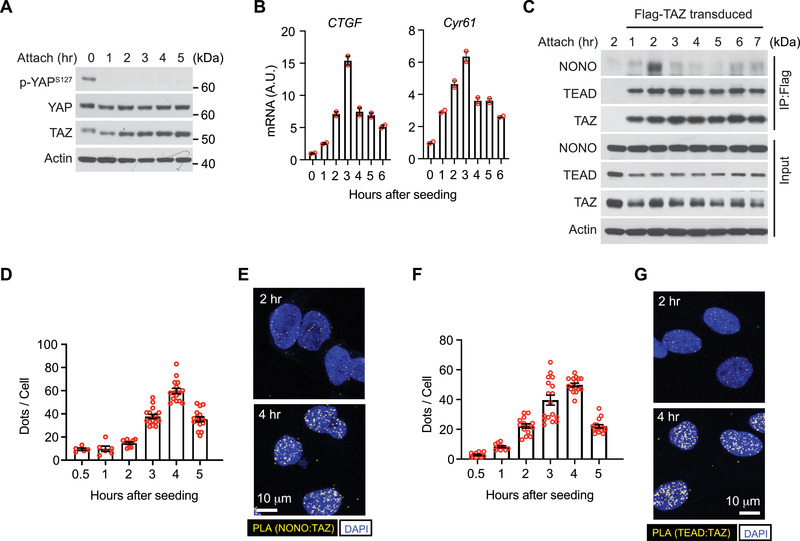
NONO interaction with TAZ is associated with TAZ activation. A) LN229 cells were seeded (attach) on Petri dishes for indicated times (in hours). Total cell lysates were subjected to western blotting. B) LN229 cells were seeded (attach) on Petri dishes for indicated hours and subjected to qPCR for *CTGF* and *Cyr61* mRNA. C) LN229 cells stably transduced with Flag‐tagged TAZ were seeded on Petri dishes for indicated hours and subjected to immunoprecipitation by a Flag antibody. The immunoprecipitated products and total lysates were subjected to western blotting. D) LN229 cells were seeded on coverslips for indicated hours and subjected to PLA using TAZ and NONO antibodies. PLA signals (dots) in each cell were quantified. Each data point represents an image field containing an average of 10 cells. *n* = 5–16 images in each condition as indicated. All images were collected from one experiment. Two independent experiments were performed and showed similar results. E) Representative results of PLA images at 2 and 4 h after seeding from (D). Scale bar = 10 µm. F) LN229 cells were seeded on coverslips for indicated hours and subjected to PLA using TAZ and TEAD antibodies. PLA signals (dots) in each cell were quantified. Each data point represents an image field containing an average of 10 cells. *n* = 10–17 images in each condition as indicated. All images were collected from one experiment. Two independent experiments were performed and showed similar results. G) Representative results of PLA images at 2 and 4 h after seeding from (F). Scale bar = 10 µm.

To examine if NONO is related to TAZ transcriptional regulatory activity, we employed a scenario in which TAZ activation can be temporally tracked. It has been previously reported that when cells are attached to a matrix, the Hippo pathway is switched off, which results in dephosphorylation and activation of YAP/TAZ.^[^ ^25^ ^]^ Consistently, we observed dephosphorylation of YAP/TAZ within one hour when LN229 cells were seeded onto a Petri dish (**Figure** **2**A). Corresponding to the dephosphorylation and activation of YAP/TAZ, expressions of *CTGF* and *CYR61*, two well characterized YAP/TAZ target genes, gradually increased upon cell attachment to dishes and peaked 3 h after seeding (Figure 2B). In this trackable system, we monitored the NONO‐TAZ interaction using immunoprecipitation. NONO coprecipitation with TAZ peaked two hours after seeding (Figure 2C). This result suggested that the NONO‐TAZ interaction is transient and likely specifically occurs when TAZ activates transcription. To further examine this notion, we used PLA to monitor the NONO‐TAZ interaction in situ at endogenous levels. PLA signals gradually increased and peaked 4 h after cell seeding (Figure 2D,E). Notably, the difference in time frames observed between the assays performed on Petri dishes (Figure 2C) and cover slips (Figure 2D) likely arose because the two surface materials have different adhesive properties for cell attachment. To examine if the time frame of PLA signals also correlates with TAZ activation, we used the TAZ‐TEAD interaction as an indicator of TAZ activation. The PLA signal from the TAZ‐TEAD interaction followed the same temporal pattern as that of TAZ‐NONO interaction (Figure 2F,G). Therefore, both of these two approaches indicated that the interaction between NONO and TAZ positively correlates with TAZ activation.

### 2.3 NONO Promotes TAZ‐Mediated Transcription

**Figure 3:**
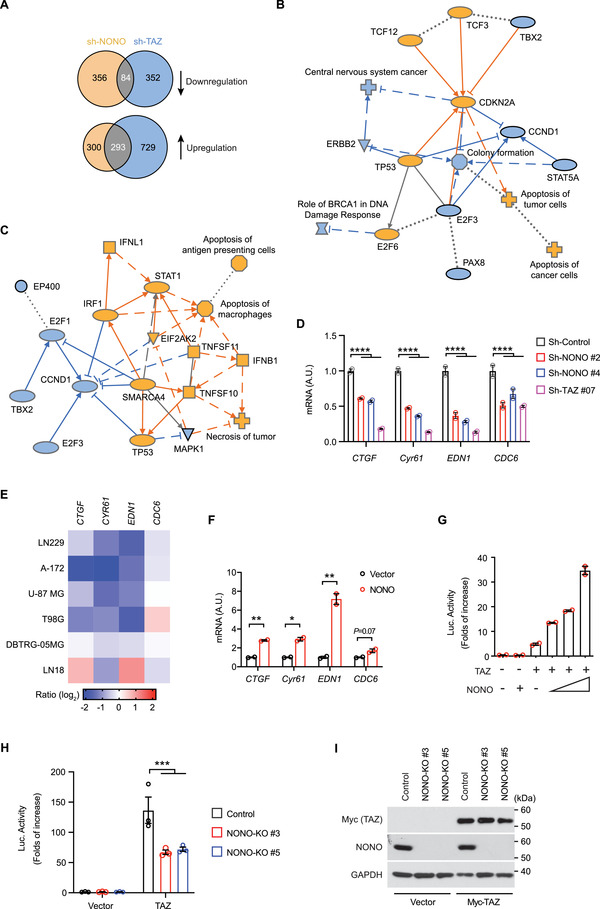
NONO promotes TAZ‐mediated transcription. A) Venn diagrams showing numbers of genes whose expression responds to shRNA‐mediated TAZ or NONO knockdown in LN229 cells. Arrows indicate downregulation or upregulation of the genes. B) Ingenuity Pathway Analysis of NONO‐regulated genes in LN229 cells predicted activation (orange) or inhibition (blue) of indicated signaling pathways or regulators. C) Ingenuity Pathway Analysis of genes coregulated by NONO and TAZ in LN229 cells predicted activation (orange) or inhibition (blue) of indicated signaling pathways or regulators. D) LN229 cells stably transduced by indicated shRNAs targeting NONO or TAZ, or a scrambled shRNA control, were subjected to q‐RT‐PCR for indicated mRNA. ****P < 0.0001. *n* = 2 biological repeats, two‐way ANOVA. E) GBM cells transfected with a pool of four siRNAs against NONO or a scrambled siRNA control were subjected to q‐RT‐PCR for indicated mRNA. Ratio of each mRNA in NONO‐depleted cells to that in control cells is shown. F) LN229 cells transfected with NONO or vector were subjected to q‐RT‐PCR for indicated mRNA. *P < 0.05, **P < 0.01. *n* = 2 biological repeats, unpaired *t*‐test.G) HEK293T cells transfected with the 8 × GTIIC‐Luc reporter, TAZ, and NONO as indicated were subjected to luciferase assay. The luciferase reading in each sample was normalized to that from cells transfected by the 8 × GTIIC‐Luc reporter alone. H) HEK293T cells with or without (control) NONO knockout transfected with the 8 × GTIIC‐Luc reporter and Myc‐TAZ as indicated were subjected to luciferase assay. The luciferase reading in each sample was normalized to that from cells transfected by the 8 × GTIIC‐Luc reporter alone in control cells. ***P < 0.001. *n* = 3 biological repeats, two‐way ANOVA. I) HEK293T cells described in (H) were subjected to western blotting.

Previous studies found that NONO can regulate gene transcription. To characterize the gene expression program regulated by NONO, we silenced NONO expression in LN229 cells by using two different shRNAs (Figure S1F, Supporting Information) and examined global gene expression by RNA‐sequencing (RNA‐seq). Integrated analysis of gene expression in response to the two shRNAs indicated that 593 and 440 genes were up‐ and down‐regulated, respectively, when NONO was knocked down (**Figure** **3**A). Ingenuity Pathway Analysis of these NONO‐regulated genes suggested that silencing NONO expression inhibits cell colony formation and promotes apoptosis (Figure 3B). In addition, the gene expression changes correlated with activation of tumor suppressors (e.g., TP53 and CDKN2A) and with inhibition of oncogenes (e.g., CCND1 and ERBB2) (Figure 3B). The gene expression program suggested that NONO may have certain tumor‐promoting properties.

To examine if there is an overlap between NONO‐ and TAZ‐regulated genes, we carried out RNA‐seq analysis of gene expression changes responding to TAZ depletion in LN229 cells. 1022 and 436 genes were up‐ and down‐regulated, respectively, when TAZ was silenced by either of two shRNAs (Figure 3A). Comparison of genes regulated by NONO or TAZ indicated that 293 and 84 genes were similarly up‐ and down‐regulated, respectively, in response to either NONO or TAZ depletion (Figure 3A). Ingenuity Pathway Analysis suggested that these gene expression changes were related to activation of cell death and inhibition of cell cycle. These processes were likely to be accompanied by activation of TP53 but inhibition of CCND1, E2F1, and MAPK1 (Figure 3C). These results suggested that NONO has a similar property to TAZ in promoting cell proliferation and inhibiting apoptosis.

To further examine if NONO regulates TAZ target genes, we first assessed *EDN1* and *CDC6*, because the above RNA‐seq analyses indicated that both of them were repressed when either NONO or TAZ was depleted, and these genes were identified to be directly regulated by the YAP/TAZ‐TEAD transcriptional machinery in cell proliferation.^[^ ^3^ ^]^ Quantitative RT‐PCR (qRT‐PCR) confirmed that *EDN1* and *CDC6* expressions are inhibited when either NONO or TAZ is depleted in LN229 cells (Figure 3D). *CTGF* and *CYR61* are well known genes directly regulated by YAP/TAZ‐TEAD. They were not among the genes coregulated by NONO and TAZ in the RNA‐seq analyses, because their expression changes were below the threshold setting. We found that their expressions were nevertheless inhibited when NONO or TAZ was depleted by shRNAs in LN229 cells (Figure 3D). To examine if the regulation is limited to LN229 cells, NONO expression was silenced by a pool of four different siRNAs in another five human glioblastoma cell lines. Among the four examined genes, expression of *CTGF*, *CYR61*, and *EDN1* were consistently repressed in five of the six examined cell lines (Figure 3E). This result suggested that regulation of these genes by NONO is not cell line‐specific and could be generalized to additional GBM cell lines. To examine if NONO is able to promote the expression of these TAZ target genes, NONO was ectopically expressed in LN229 cells. Compared to cells transfected with empty vector, cells expressing recombinant NONO showed higher expression of these genes (Figure 3F). To further examine this notion more directly, we utilized the 8 × GTIIC‐Luc reporter, a well‐characterized reporter of YAP/TAZ activity.^[^ ^26^ ^]^ As expected, ectopic expression of TAZ by itself increased the reporter activity in 293T cells (Figure 3G). Expression of NONO further enhanced the reporter activity in a dosage‐dependent manner (Figure 3G). Conversely, in NONO knockout cells, expression of TAZ has less capability to promote the reporter activity compared to the wild‐type cells (Figure 3H,I). These results indicated that NONO is able to promote TAZ's capability in driving transcription. Overall, these results supported that NONO is involved in promoting TAZ transcriptional activity.

### 2.4 NONO is Required for TAZ to Access Transcriptional Enhancers

**Figure 4:**
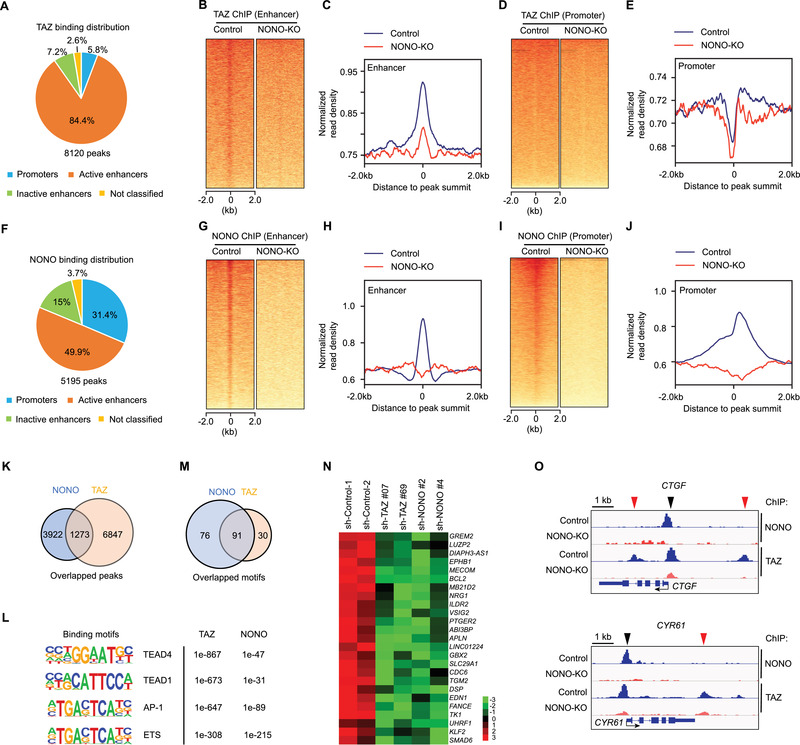
NONO is required for TAZ to access transcriptional enhancers. A) Fraction of TAZ peaks associated with each transcriptional regulatory element category. B) Heatmap showing TAZ‐binding sites located on enhancers derived from analyzing TAZ ChIP‐seq data in NONO WT or knocked out (KO) LN229 cells. C) Intensity distribution of TAZ‐binding sites around the binding enhancer peaks from the analysis shown in (A). D) Heatmap showing TAZ‐binding sites located on transcription start sites (TSS) derived from analyzing TAZ ChIP‐seq data in NONO WT or knocked out (KO) LN229 cells. E) Intensity distribution of TAZ‐binding sites around the binding TSS peaks from the analysis shown in (C). F) Fraction of NONO peaks associated with each transcriptional regulatory element category. G) Heatmap showing NONO‐binding sites located on enhancers derived from analyzing NONO ChIP‐seq data in NONO WT or knocked out (KO) LN229 cells. H) Intensity distribution of NONO‐binding sites around the binding enhancer peaks from the analysis shown in (E). I) Heatmap showing NONO‐binding sites located on transcription start sites (TSS) derived from analyzing NONO ChIP‐seq data in NONO WT or knocked out (KO) LN229 cells. J) Intensity distribution of NONO‐binding sites around the binding TSS peaks from the analysis shown in (G). K) Overlap of peaks from TAZ or NONO ChIP‐seq analysis. L) Top enriched transcription factor‐binding motifs associated with TAZ or NONO peaks. p‐values for each corresponding enrichment were shown. M) Overlap of transcription factor‐binding motifs associated with TAZ or NONO peaks. N) Genes, whose expression was decreased when either TAZ or NONO was knocked down by indicated shRNAs, were surveyed for TAZ and NONO peaks at their transcription promoters. The genes shown here are those having TAZ and NONO peaks at these regions. Expression of these genes (assessed by RNA‐seq) was shown. The scale shows log2 fold change. O) TAZ or NONO peaks associated with the genomic loci flanking CTGF or CYR61 gene in LN229 cells with (NONO‐KO) or without (Control) NONO knockout. The transcription starting sites and orientation for each gene were indicated by arrows.

To understand how NONO promotes TAZ‐mediated transcription, we hypothesized that NONO may regulate TAZ to access the genomic sites where it promotes gene expression. Previous studies found that TAZ and YAP are mostly associated with transcription enhancers, but not promoters, to drive oncogenic growth.^[^ ^3^, ^4^ ^]^ We therefore performed chromatin immunoprecipitation sequencing (ChIP‐seq) experiments in LN229 cells to examine if the association of TAZ with enhancers requires NONO. Three transcription‐related epigenetic markers—histone H3 lysine 4 monomethylation (H3K4me1), histone H3 lysine 4 trimethylation (H3K4me3), and histone H3 lysine 27 acetylation (H3K27Ac)—were used as indicators of enhancers and promotors. We observed that most (84.4%) of TAZ peaks are associated with active enhancers (H3K4me1^+^, H3K4me3^−^, H3K27Ac^+^), whereas a small proportion of TAZ peaks locate at inactive enhancers (H3K4me1^+^, H3K4me3^−^, H3K27Ac^−^) or promoters (H3K4me1^−^, H3K4me3^+^, H3K27Ac^+^) (**Figures** **4**A–E, results in control cells). This observation is consistent with the previous observation that TAZ is largely associated with enhancers.^[^ ^3^ ^]^ Interestingly, loss of NONO markedly reduces TAZ distribution in the enhancer regions (Figure 4B,C), suggesting that NONO is important for TAZ association to the enhancers. Next, to examine if NONO is similarly associated with enhancers, we performed NONO ChIP‐seq experiments. Indeed, the largest portion (49.9%) of NONO peaks are associated with active enhancers, while 31.4% of NONO peaks are at promoters (Figure 4F). In NONO knocked out (NONO‐KO) cells, the peaks from NONO ChIP‐seq associated with enhancers or promoters were eliminated (Figure 4G–J), therefore confirming the specificity of the peaks seen in control cells. Comparison of the genomic loci associating with TAZ and that with NONO indicated that 24.5% (1273 out of 5195) of NONO‐associating loci overlapped with 15.7% (1273 out of 8120) of TAZ‐associating loci (Figure 4K). The *de novo* motif analyses revealed that TAZ peaks are mostly enriched by binding motifs of the well‐known TAZ‐associating transcriptional factors, such as the AP1 and TEAD transcription factor families (Figure 4L; Table S2, Supporting Information), and that binding motifs of the ETS transcription factor family are more enriched in NONO peaks (Figure 4L; Table S3, Supporting Information). Remarkably, 75.2% (91 out of 121) of the enriched motifs, such as AP1 and TEAD, in TAZ peaks overlap with those in NONO peaks (Figure 4M; Table S4, Supporting Information), suggesting a strong involvement of NONO in TAZ‐regulated transcription. Notably, because many NONO‐associating loci do not overlap with TAZ‐associating loci, it is possible that NONO has TAZ‐independent functions.

With these genomic loci coassociated by TAZ and NONO, we assessed if genes whose expression is coregulated by TAZ and NONO are bound by them. The analysis revealed that both TAZ and NONO can access the promoters of 22.9% (25 out of 84) of genes coactivated by them and 13.6% (46 out of 293) genes corepressed by them (Figure 4N; Figure S4A, Supporting Information). Therefore, these genes are likely directly coregulated by TAZ and NONO. Since *CTGF* and *CYR61* are well characterized TAZ target genes and our previous studies showed that they can also be coactivated by TAZ and NONO (Figure 3D–F), we examined the genomic loci of these two genes. The ChIP‐seq data indicated that both TAZ and NONO have a strong binding peaks close to the transcription starting sites (TSS) of *CTGF* and *CYR61* genes (Figure 4O, black arrow heads). In NONO‐KO cells, both TAZ and NONO peaks were eliminated, suggesting that TAZ association with these genes at these sites depends on NONO. Notably, there are additional TAZ peaks within 4 kb upstream or downstream of the gene TSS (Figure 4O, red arrow heads). In NONO‐KO cells, these additional peaks were also eliminated although there were no corresponding NONO peaks observed at these loci. These results suggested that NONO could also promote TAZ association to certain genomic loci when NONO is not directly associated with these loci. Overall, these results suggested that NONO is involved in facilitating TAZ to access transcriptional enhancers as well as promoters of the target genes.

### 2.5 NONO is Required for TAZ to Access TEAD and Rpb1

**Figure 5:**
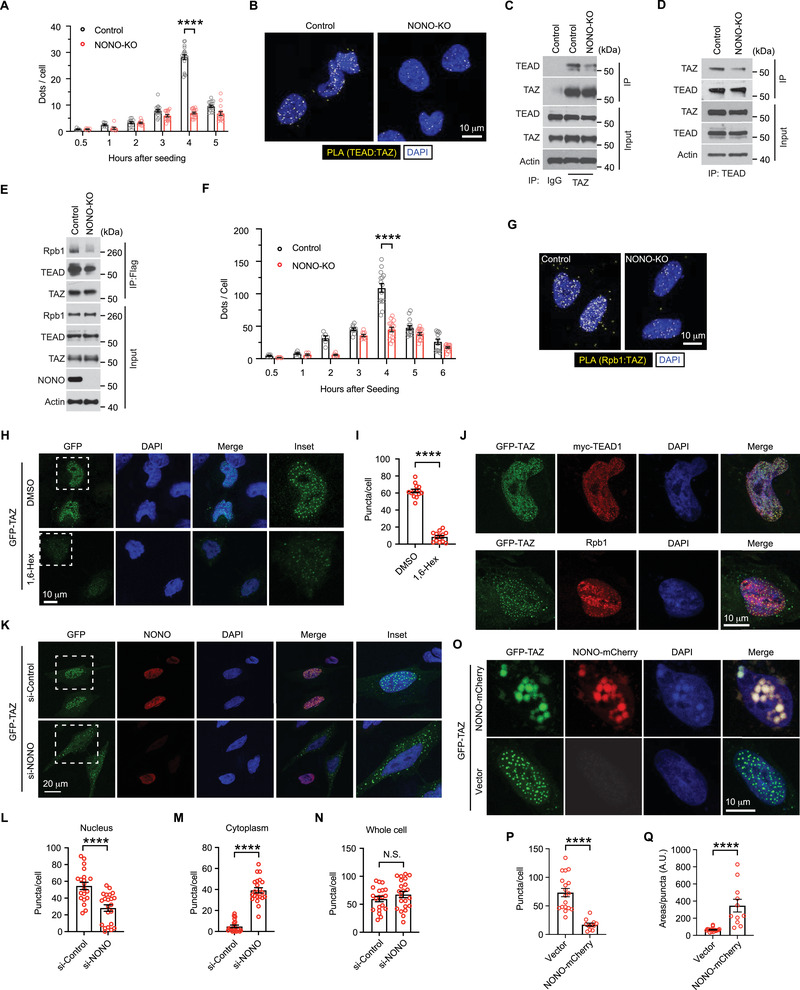
NONO is required for TAZ to access TEAD and Rpb1. A) LN229 cells, in which NONO was or was not knocked out (KO), were seeded on coverslips for indicated hours and subjected to PLA using TAZ and TEAD antibodies. PLA signals (dots) in each cell were quantified. Two‐way ANOVA. ****P < 0.0001. Each data point represents an image field containing an average of 10 cells. *n* = 6–20 images in each condition as indicated. All images were collected from one experiment. Two independent experiments were performed and showed similar results. B) Representative results of PLA images at 4 h after seeding from (A). C,D) LN229 cells, in which NONO was or was not knocked out (KO), were seeded on Petri dishes for 2 h and subjected to immunoprecipitation by a TAZ antibody or C) IgG control, or D) TEAD antibody. The immunoprecipitated products were subjected to western blotting. E) LN229 cells stably transduced by Flag‐tagged TAZ were seeded on Petri dishes for 2 h and subjected to immunoprecipitation by a Flag antibody. The immunoprecipitated products and total lysates were subjected to western blotting. F) LN229 cells, in which NONO was or was not knocked out (KO), were seeded on coverslips for indicated hours and subjected to PLA using TAZ and Rpb1 antibodies. PLA signals (dots) in each cell were quantified. Two‐way ANOVA. ****P < 0.0001. Each data point represents an image field containing an average of 10 cells. *n* = 5–15 images in each condition as indicated. All images were collected from one experiment. Two independent experiments were performed and showed similar results. G) Representative results of PLA images at 4 h after seeding from (F). H) LN229 cells transiently transfected by GFP‐TAZ were treated by DMSO or 2% 1, 6‐hexanediol for 2 min and subjected to fluorescence imaging by confocal microscopy. Outlined areas are enlarged and shown in the insets. I) GFP‐TAZ puncta in each cell from the experiment described in (H) were quantified. Unpaired student *T*‐test. ****P < 0.0001. Each data point represents a cell with certain levels of GFP signal. *n* = 14 cells in each condition. All cell images were collected from three independent experiments. J) LN229 cells transiently transfected by GFP‐TAZ alone (lower) or with myc‐TEAD1 (upper) were subjected to immunofluorescent staining. K) LN229 cells transfected by a pool of four siRNAs against NONO (si‐NONO) or a scrambled siRNA control were transiently transfected by GFP‐TAZ and subjected to immunofluorescent staining. Outlined areas are enlarged and shown in the insets. (L‐N) GFP‐TAZ puncta in the L) cell nucleus, M) cytoplasm, or N) whole cell of each cell from the experiment described in (K) were quantified. Unpaired student *T*‐test. ****P < 0.0001. N.S., *P* > 0.05. Each data point represents a cell with certain levels of GFP signal. n_si‐control_ = 20, n_si‐NONO_ = 22 cells in each condition. All cell images were collected from three independent experiments. O) GFP‐TAZ was cotransfected with NONO‐mCherry or vector into LN229 cells. The cells were then fixed and subjected to fluorescence imaging by confocal microscopy. P) GFP‐TAZ puncta number or Q) size in each cell from the experiment described in (O) were quantified. Unpaired student *T*‐test. ****P < 0.0001. Each data point represents a cell with certain levels of GFP signal. n_vector_ = 23, n_Nono‐mCherry_ = 19 cells in each condition. All cell images were collected from three independent experiments.

As a transcriptional coactivator, TAZ accesses DNA largely through binding to TEAD family of transcription factors. Because the TAZ‐TEAD interaction showed similar kinetics to the TAZ‐NONO interaction (Figure 2D–G), we examined if NONO is involved in promoting the interaction between TAZ and TEAD. Knocking out NONO in LN229 cells markedly reduced the PLA signal generated by the TAZ‐TEAD interaction at 4 h after cell attachment (**Figure** **5**A,B), suggesting the enhanced TAZ‐TEAD interaction responding to cell attachment is promoted by NONO. To further test this notion, we conducted co‐immunoprecipitation of endogenous TAZ and TEAD. As expected, the reciprocal precipitation of each other in between TAZ and TEAD in LN229 cells was reduced when NONO was knocked out (Figure 5C,D). In addition, when NONO was knocked down by a pool of four siRNAs, TEAD coprecipitated with recombinant TAZ in LN229 cells was also reduced (Figure S5A, Supporting Information). These results further support that NONO is involved in promoting the TAZ‐TEAD interaction.

Previous studies demonstrated that NONO activates transcription by coupling several transcription activators to RNA polymerase II (RNAP II)^[^ ^27^, ^28^ ^]^ via direct binding to the carboxyl‐terminal domain (CTD) of RNAP II by NONO.^[^ ^29^ ^]^ We wondered if NONO could facilitate the access of TAZ to RNAP II and thereby activate transcription. PLA analyses indicated that both NONO and TAZ are able to interact with Rpb1 in the nucleus of LN229 cells (Figure S5B,C, Supporting Information). The interaction between TAZ and Rpb1 was further supported by the observation that Rpb1 can be coprecipitated by recombinant TAZ from LN229 cells (Figure 5E). Interestingly, PLA showed that the TAZ‐Rpb1 interaction followed the same temporal pattern as that of TAZ‐NONO and TAZ‐TEAD interactions (Figure 5F, comparing to Figure 2D–G). In NONO knockout cells, the TAZ‐Rpb1 interaction was reduced, especially at 4 h after cell attachment, when the TAZ‐Rpb1 interaction peaked in control cells (Figure 5F,G). The reduction of the TAZ‐Rpb1 interaction in NONO‐depleted cells was also observed by the immunoprecipitation assay (Figure 5E). Notably, NONO knockout did not affect the phosphorylation of Lats1 and TAZ, nor did it decrease TAZ expression (Figure S5D, Supporting Information). Therefore, the reduction of TAZ‐TEAD and TAZ‐Rpb1 interactions is unlikely due to a change of TAZ regulation by Lats1. These results supported that NONO is involved in facilitating access of TAZ to TEAD and Rpb1.

### 2.6 NONO Promotes TAZ to Form LLPS Condensates in the Nucleus

TAZ can form liquid–liquid phase separation (LLPS) condensates.^[^ ^6^ ^]^ When occurring in the nucleus, LLPS was proposed to promote its ability to regulate gene expression through inducing TEAD and other transcription cofactors to condensate into LLPS domains.^[^ ^6^ ^]^ Since NONO regulates TAZ transcriptional activity and its interaction with TEAD, Rpb1, as well as enhancers, we wondered if NONO may affect TAZ LLPS. First, we examined TAZ LLPS in LN229 cells. EGFP‐tagged TAZ (EGFP‐TAZ) expressed in LN229 cells formed multiple puncta in the nucleus. The puncta number was markedly reduced by 2% 1, 6‐hexanediol (Figure 5H,I). Staining of TEAD or Rpb1 in these cells indicated that EGFP‐TAZ puncta overlap with TEAD and —to a lesser extent— Rpb1 puncta in the nucleus (Figure 5J). Similar nuclear EGFP‐TAZ puncta were also observed in HEK293T cells (Figure S5E, Supporting Information). These results are consistent with previous observations in other cells^[^ ^6^ ^]^ and suggested occurrence of TAZ LLPS in these cells. We then examined whether TAZ LLPS can be regulated by NONO. In both LN229 and HEK293T cells, depletion of NONO significantly reduced the number of EGFP‐TAZ puncta in the nucleus (Figure 5K,L; Figure S5F,G, Supporting Information). Surprisingly, more EGFP‐TAZ puncta were found in cytoplasm of NONO‐depleted cells than control cells, while the total EGFP‐TAZ puncta number in whole cells did not change (Figure 5K,M,N; Figure S5F,G, Supporting Information). Because TAZ phosphorylation by Lats1/2 can induce its nuclear exclusion, we examined whether NONO‐depletion may affect TAZ phosphorylation. EGFP‐TAZ expressed in NONO knockout HEK293T cells does not show more phosphorylation than the control cells (Figure S5H, Supporting Information), suggesting that the localization change of TAZ LLPS is unlikely due to the change of TAZ phosphorylation. These results suggested that NONO is specifically required for TAZ LLPS in the nucleus. To further examine the capability of NONO in regulating TAZ LLPS, mCherry‐tagged NONO (NONO‐mCherry) was coexpressed with EGFP‐TAZ. These two proteins formed overlapped puncta in the nucleus (Figure 5O; Figure S5I, Supporting Information). Comparing to EGFP‐TAZ expressed alone, the EGFP‐TAZ puncta in NONO‐mCherry expressing cells are much larger, albeit fewer (Figure 5O,P,Q; Figure S5I,J, Supporting Information). These results indicated that NONO can promote TAZ LLPS in the nucleus. Overall, the above studies indicated that NONO is involved in facilitating TAZ LLPS in the nucleus.

### 2.7 NONO Expression is Upregulated in GBM and Associates with Poor Survival

**Figure 6:**
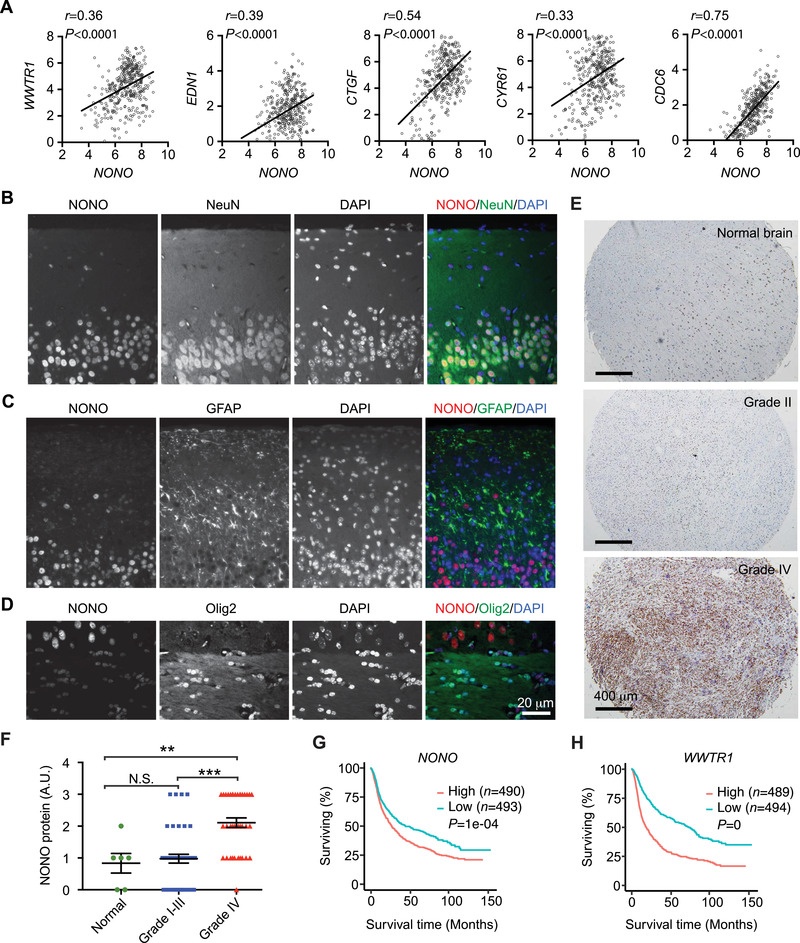
NONO expression is higher in GBM and associates with poor survival. A) The expression correlations of NONO with indicated genes were analyzed in the CGGA GBM dataset (*n* = 386) through GlioVis. Pearson correlation coefficients were calculated. B–D) Formaldehyde‐fixed paraffin‐embedded normal mouse brain sections were subjected to immunofluorescence staining for NONO and each indicated protein. Scale bar = 20 µm. E) Chromogenic immunodetection of NONO in formaldehyde‐fixed paraffin‐embedded normal human brain and human glioma sections. Scale bar = 400 µm. F) Quantification of NONO expression in the stained tissue sections from (E). *n*_Normal brain_ = 6, *n*_Grade I‐III glioma_ = 43, *n*_Grade IV glioma_ = 37, **P < 0.01, ***P < 0.001, N.S., Non‐significant. Ordinary one‐way ANOVA. G,H) Kaplan‐Meier curves of patients showing G) higher or lower NONO or H) WWTR1 mRNA expression. Analysis was through GiloVis using the CGGA dataset including all tumor types, cutoff: median. Log‐rank test. *n* indicates number of human subjects.

To investigate the functional association between NONO and TAZ, we examined NONO expression in clinical GBM. Analysis of the CGGA GBM dataset^[^ ^30^ ^]^ revealed that NONO expression positively correlates with TAZ (**Figure** **6**A). Likewise, we observed a similar positive correlation in expression of NONO and the four TAZ target genes, including *EDN1, CTGF, CYR61*, and *CDC6*. This result further supported our observation in vitro (Figure 2D–F) that expressions of these genes can be upregulated by NONO. To further examine NONO expression in gliomas, we performed immunohistochemistry using a NONO monoclonal antibody. The specificity of this antibody was confirmed with a tumor section in which NONO was knocked down (Figure S6A, Supporting Information). In normal mouse brain sections, NONO was mostly expressed in neurons, which were identified by the neuronal marker, NeuN (Figure 6B). However, in areas enriched with astrocytes (identified by the astrocyte marker, GFAP) or with oligodendrocytes (identified by the oligodendrocyte marker, Olig2), NONO was expressed at much lower levels (Figure 6C,D). These results were consistent with previous studies,^[^ ^31^ ^]^ and indicated that NONO protein expression is lower in non‐neoplastic glial cells. We then examined NONO in glioma patient samples (Grade I‐III, *n* = 43; Grade IV, *n* = 37) and normal human brain samples (*n* = 6). We found that NONO protein expression in grade IV gliomas is significantly higher than in lower grade gliomas and normal brains (Figure 6E,F). We also examined if NONO expression in gliomas correlates with patient survival. By analyzing the CGGA dataset using GlioVis (http://gliovis.bioinfo.cnio.es/), we found that higher NONO expression correlates with shorter survival in GBM patients (Figure S6B, Supporting Information). Similarly, higher TAZ expression also predicts poor prognosis. When analyzing the cohort of patients bearing gliomas at various grades, the correlation between NONO or TAZ expression with poor survival was more remarkable (Figure 6G,H). Overall, the above results suggested that NONO and TAZ may function synergistically to promote GBM tumorigenesis and tumor aggressiveness.

### 2.8 NONO is Important for TAZ‐Driven Oncogenicity of Glioma Cells

**Figure 7:**
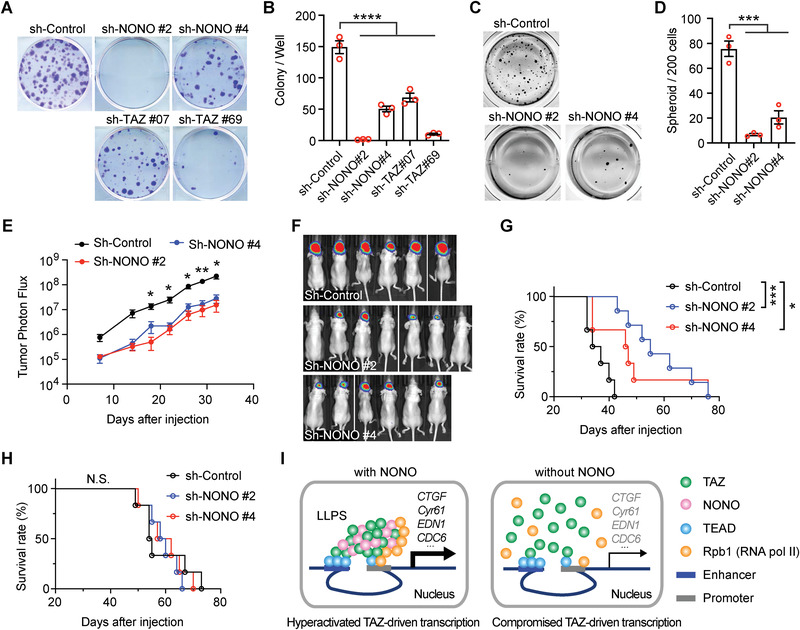
NONO is important for TAZ‐driven oncogenicity of glioma cells. A) LN229 cells stably transduced by indicated shRNAs targeting NONO or TAZ, or a scrambled shRNA control, were subjected to the colony formation assay. B) Colony numbers in each well from the results shown in (A) were counted. *n* = 3, Ordinary one‐way ANOVA, ****P < 0.0001. C) LN229 cells stably transduced with TAZ^4SA^ and indicated shRNAs targeting NONO or a scrambled shRNA control were subjected to the tumor sphere assay. D) Sphere numbers in each well from the results shown in (C) were counted. *n* = 3, ***P < 0.001. Ordinary one‐way ANOVA. E) LN229 cells stably transduced with TAZ^4SA^ and indicated shRNAs targeting NONO or a scrambled shRNA control were intracranially implanted into nude mice. Tumor photon flux from bioluminescence imaging of mice implanted with these cells is shown. *n*_sh‐Control_ = 6 mice, *n*_sh‐NONO#2_ = 7 mice, *n*_sh‐NONO#4_ = 6 mice, *P* values of the comparison between sh‐Control and each of the sh‐NONO at each time point are indicated. *P < 0.05, **P < 0.01. Two‐way ANOVA. F) Bioluminescence images of mice in the experiment shown in (E) at day 32 after injection. G) Kaplan‐Meier survival curves of mice implanted with LN229 cells stably transduced with TAZ^4SA^ and indicated shRNAs targeting NONO or a scrambled shRNA control. *n*_sh‐Control_ = 6 mice, *n*_sh‐NONO#2_ = 7 mice, *n*_sh‐NONO#4_ = 6 mice, *P < 0.05, ***P < 0.001. Log‐rank test. H) Kaplan‐Meier survival curves of mice implanted with LN229 cells stably transduced with empty vector control and indicated shRNAs targeting NONO or a scrambled shRNA control. *n* = 6 mice for each group. N.S. (no significance), *P* > 0.05. Log‐rank test. (I) A proposed model showing how NONO is involved in activating TAZ transcriptional activities.

To examine if NONO is involved in promoting oncogenic properties of glioma cells, its expression was silenced by either of two different shRNAs. Knocking down NONO inhibited the clonogenicity of LN229 cells in the two‐dimensional colony formation assay (**Figure** **7**A,B). This inhibitory effect was also observed when TAZ expression was silenced by shRNAs. In addition, when NONO expression was silenced in U‐87MG or LN18 glioblastoma cells, the clonogenicity was also reduced (Figure S7A,B, Supporting Information), indicating the growth inhibitory effect is not limited to LN229 cells. To examine if NONO is important for TAZ‐driven oncogenic properties, we stably expressed the TAZ^4SA^ constitutive mutant,^[^ ^32^ ^]^ which is able to promote tumorigenesis of LN229 cells.^[^ ^13^ ^]^ Depletion of NONO by the two shRNAs in LN229 cells expressing TAZ^4SA^ (denoted as LN229^TAZ(4SA)^) inhibited sphere formation in the three‐dimensional neural sphere assay (Figure 7C,D), indicating that the oncogenicity of LN229^TAZ(4SA)^ also depends on NONO. To further test this notion, we employed an orthotopic mouse GBM model. In this model, expression of TAZ^4SA^ in LN229 cells resulted in more aggressive tumors, thereby shortening the survival of mice (comparing the sh‐controls in Figure 7G,H).^[^ ^13^ ^]^ Silencing the expression of NONO in LN229^TAZ(4SA)^ cells significantly reduced the growth of these tumors (Figure 7E,F). In addition, mice bearing the NONO‐depleted LN229^TAZ(4SA)^ tumors showed prolonged survival compared to those bearing control tumors (Figure 7G). Interestingly, mice bearing tumors derived from NONO‐depleted LN229 cells did not show prolonged survival compared to those bearing LN229 tumors transduced with the scrambled control shRNA (Figure 7H). Such differential effects on survival suggested that TAZ‐driven tumor aggressiveness directly depends on NONO. Overall, the above results supported that NONO is involved in promoting TAZ activity to drive GBM tumorigenesis and aggressiveness.

## 3 Discussion

In this study, we found that NONO is a TAZ‐binding protein in the nucleus. The binding between TAZ and NONO has similar temporal kinetics to those of TAZ interaction with TEAD and Rpb1, as well as to those of the expression of TAZ target genes. Such correlation suggests that NONO plays a role in TAZ activation. Consistently, depletion of NONO reduces the transcription of TAZ target genes. Mechanistically, we found that NONO promotes the interaction between TAZ and TEAD as well as between TAZ and Rpb1, thereby allowing the access of TAZ to its genomic targets. Furthermore, we showed that NONO is responsible for TAZ to form LLPS condensates in the nucleus. Because LLPS has recently been proposed to be a way for TAZ to activate transcription by compartmentalizing key cofactors,^[^ ^6^ ^]^ our results suggested that NONO is involved in promoting TAZ function by facilitating its LLPS. In the study, we further found that expressions of NONO and TAZ are both upregulated in human GBM and predict worse survival. Silencing NONO expression inhibits TAZ‐driven tumorigenesis. Overall, this study revealed that NONO is a nuclear factor promoting TAZ LLPS and activation in driving the oncogenic transcriptional program (Figure 7I).

Compared to the many known cytoplasmic binding partners of YAP and TAZ, few nuclear binding partners have been identified so far.^[^ ^33^ ^]^ This leaves a question as to whether nuclear YAP and TAZ are active by default. Recent studies suggested that their activities could still be regulated even in the nucleus. The SWI/SNF complex is able to regulate the ability of YAP/TAZ to access TEAD and their target genes.^[^ ^23^, ^34^, ^35^ ^]^ In addition, YAP and TAZ can form phase‐separated nuclear condensates, which promote their ability to regulate gene expression.^[^ ^6^, ^7^ ^]^ Here, we observed that the TAZ‐TEAD and TAZ‐Rpb1 interactions are transient. This suggests that their interactions could be dynamically regulated after TAZ enters the nucleus. Such regulation may provide another safeguard mechanism in addition to cytosolic regulation to keep TAZ‐mediated oncogenic transcription programs under control. While investigating how such transient interactions were achieved, we found that NONO is required for TAZ to maximize its interactions with TEAD and Rpb1. This suggests that NONO may be involved in promoting the transient interaction. Consistent with this notion, the temporal kinetics of NONO interaction with TAZ are similar to that of interactions between TAZ and the other two proteins. This may be because NONO is responsible for recruiting them into certain nuclear domains, thereby enhancing their interaction. In line with this notion, TAZ can form LLPS condensates in the nucleus (Figure 5H; Figure S5E, Supporting Information)^[^ ^6^ ^]^ and NONO appears to be required for the process (Figure 5K; Figure S5F, Supporting Information). Therefore, NONO may promote TAZ interaction with TEAD and Rpb1 though facilitating TAZ LLPS. It was known that NONO and other DBHS proteins as well as the long noncoding RNA NEAT1 form paraspeckles in the nucleus.^[^ ^14^ ^]^ Although the functions of this nuclear structure are unclear, one function might be gene expression regulation by sequestering proteins or mRNAs.^[^ ^36^ ^]^ Whether paraspeckles are a type of LLPS condensates and could be involved in the formation of YAP or TAZ nuclear condensates is a question that warrants future investigation.

Our study indicated that NONO preferentially interacts with TAZ over YAP. The motif of TAZ involved in the interaction with NONO is between aa 120–164, which contains the WW motif. Because TAZ interacts with many other proteins by using this WW motif to bind to the PPxY motif on the other proteins, we have examined a TAZ WW motif mutation. Interestingly, the WW mutation does not disrupt the binding between NONO and TAZ (data not shown). In addition, there is no PPxY motif in NONO. Therefore, it is unlikely that the interaction between TAZ and NONO occurs through the WW‐PPxY binding module. Non‐canonical protein‐protein interaction modules may be involved in mediating the interaction between NONO and TAZ. Alternatively, TAZ and NONO may interact with each other indirectly through a mediator. Both of these possibilities may distinguish TAZ from YAP in interacting with NONO, thus explaining why NONO appears to have a weaker interaction with YAP. Nevertheless, whether NONO can also regulate YAP in a similar way to TAZ warrants further study.

NONO is ubiquitously expressed in most tissues.^[^ ^15^ ^]^ However, NONO‐deficient mice develop normally,^[^ ^37^, ^38^ ^]^ except for showing a smaller cerebellum and some cognitive and affective deficits.^[^ ^31^ ^]^ These studies suggested that, in normal physiological conditions, NONO might be dispensable, or its functions could be compensated by other DBHS proteins with redundant functions. In some cancers, such as melanoma,^[^ ^16^ ^]^ breast cancers,^[^ ^17^ ^]^ and neuroblastoma,^[^ ^18^ ^]^ NONO expression is upregulated. Our study showed that NONO expression is similarly upregulated in GBM and correlates with increased malignancy. These observations suggested that NONO possesses tumor‐promoting functions, and they are therefore in line with the observations in other cancers. Our studies showed that NONO depletion has a direct inhibitory effect on hyperactivated TAZ‐driven GBM tumor growth (Figure 7G,H). This result suggests that hyperactivated TAZ‐driven GBM aggressiveness depends on NONO in order to activate oncogenic transcriptional programs. Since NONO appears to be nonessential in normal physiology,^[^ ^37^, ^38^ ^]^ the regulation of TAZ by NONO may be exploited for therapeutics of TAZ‐driven MES GBM.

## 4 Experimental Section

### Human GBM Sample Analysis

Brain glioblastoma tissue arrays with normal brain tissue as control (GL806d, containing 40 cases) and brain tumor tissue array with normal tissue (GL482, containing 48 cases) were purchased from Biomax.us. Pathology diagnosis classifications and case profiles were provided in the specification sheet of each tissue array. Tumor grade was also examined by a trained researcher. Malignant tumors were defined by high‐grade features including mitotic figures, vascular proliferation, and pseudopalisading necrosis. To examine NONO protein expression in these tissue arrays, chromogenic immunodetection was performed (method further described below). Images of the stained samples were acquired with an Olympus CX41 microscope UPLFLN 4x objective lens. For each patient sample, an image covering approximately 80% of each tissue sample was acquired. The NONO protein signal in the image was scored in a semiquantitative manner as follows: 0 (absent), 1 (0–25%), 2 (25‐75%), 3 (75‐100%).

### Mice

The tumorigenesis experiments were performed by following the previous procedure.^[^ ^13^ ^]^ Briefly, six‐ to eight‐week‐old female athymic nude mice (*Nu(NCr)‐Foxn1nu*, from Charles River, Strain Code: 490) were used. LN229 cells were first transduced with a retroviral vector expressing firefly luciferase. These cells were then transduced with retroviral or lentiviral vectors expressing the indicated shRNAs or cDNAs. For each mouse, 3  ×  10^5^ cells were injected into the right hemisphere at coordinates (+1, +2, ‐3). Brain tumor growth was monitored with bioluminescence using the IVIS imaging system (Xenogen, Alameda, CA). Photons emitted from the brain region were quantified using Living Image software (Xenogen). Luciferase activity was measured as previously descried,^[^ ^39^ ^]^ with data presented as the photons emitted per second. All animals were housed in a room with a 12‐hour light/dark cycle, free access to a standard rodent diet and water, ambient temperature maintained between 18–23 degrees Celsius, and humidity maintained between 40–60%. All experiments described in this study were carried out with the approval of the Penn State University Institutional Animal Care and Use Committee and in accordance with its guidelines.

### Cells

Human GBM cell lines—LN229 (CRL‐2611), U‐87 MG (HTB‐14), LN18 (CRL‐2610), A‐172 (CRL‐1620), T98G (CRL‐1690), and DBTRG‐05MG (CRL‐2020)—were purchased from ATCC. HEK293T cell line was purchased from Invitrogen (#R70007). These cells were cultured in Dulbecco's modified Eagle's medium (DMEM; 10‐013‐CV, Corning) supplemented with 10% fetal bovine serum (FBS; Gibco, 10 437 028) and 1% Antibiotic–Antimycotic Solution (30‐004‐CI, Corning) at 37 °C with 5% CO_2_. None of these cell lines were listed in the database of misidentified cell lines maintained by ICLAC and NCBI Biosample. These cell lines were not authenticated in this study. All cell lines were confirmed as *Mycoplasma* negative before experiments. Unless otherwise indicated, cells were grown to 50% confluence.

### Immunoprecipitation of Flag‐Tagged Proteins

LN229 or HEK293T cells expressing indicated recombinant proteins were rinsed in ice‐cold PBS and lysed in RIPA buffer (50 mM Tris, pH 7.5, 150 mM NaCl, 4 mM EDTA, 1 mM EGTA, 1% Triton X‐100, 0.5% sodium deoxycholate, 0.1% sodium dodecyl sulfate, 10% glycerol, 1x phosphatase inhibitor (Roche), and 1x proteinase inhibitor (Roche)) or RIPA buffer without SDS as indicated. Cell extracts were incubated with benzonase nuclease (Sigma #E1014) at 4 °C for 30 min. After spinning down, the supernatants were incubated with anti‐FLAG M2 affinity gel (Sigma) at 4 °C for 2 h. The beads were washed for three times with IP2 buffer (50 mM Tris, pH 7.5, 150 mM NaCl, 4 mM EDTA, 1 mM EGTA, 0.5% NP‐40, 10% glycerol, 1x phosphatase inhibitor (Roche) and 1x proteinase inhibitor (Roche)) and eluted with 3xFLAG Peptide (Sigma # F4799) dissolved in TBS (10 mM Tris HCl, 150 mM NaCl, pH 7.4). The proteins were then prepared and separated on 4–12% Bis‐Tris SDS‐PAGE gels (Invitrogen) and subjected to standard immunoblotting.

### Immunoprecipitation of Endogenous Protein

3 × 10^6^ LN229 cells were seeded in a 10‐cm plate for 2 h, lysed and then incubated with benzonase nuclease in RIPA buffer as described above. Cell extracts were collected by centrifugation, incubated with anti‐TAZ (CST, #4883) antibody or rabbit IgG (Sigma‐Aldrich, #I5006) at 4 °C overnight, and further incubated with protein A/G agarose beads (ThermoFisher, #20 423) for 2 h. The precipitates were washed with RIPA buffer three times. Bound proteins were dissociated in 20 µl of SDS sample buffer (25 mM Tris, pH 6.8, 4% SDS, 5% Glycerol, and bromophenol blue). The proteins were separated on 4–12% Bis‐Tris SDS‐PAGE gels (Invitrogen) and subjected to standard immunoblotting.

### GST Pull Down Assay

GST‐RRM(NONO) was expressed in *E.coli* through the pGEX‐T2‐GST vector. The bacteria were lysed by sonication in cold PBS lysis buffer (1X PBS, 1% Triton X‐100, 1X proteinase inhibitor, and 1 mg/ml lysozyme). The lysates were briefly collected by centrifugation and subjected to purification with glutathione agarose (ThermoFisher, #16 100). After washing with PBS washing buffer (1X PBS, 1% Triton X‐100, and 1X proteinase inhibitor) three times, the agarose with bound proteins (GST‐RRM beads) was stored in 200 µl PBS with protease inhibitors at 4 °C. HEK293T cells were lysed in RIPA buffer as described above and incubated with 10 µl GST‐RRM beads as obtained above overnight at 4 °C. After being washed with RIPA buffer three times, bound proteins on the beads were dissociated in 20 µl of SDS sample buffer and subjected to standard immunoblotting as described above.

### Immunoblotting

Immunoblotting was conducted as follows.^[^ ^39^ ^]^ Briefly, cells were lysed in SDS lysis buffer (10 mM Tris pH 7.5, 1% SDS, 50 mM NaF, and 1 mM NaVO_4_) and subjected to SDS‐PAGE on 4–12% Bis‐Tris SDS‐PAGE gels (Invitrogen) and transferred to Immobilon‐P membranes (Millipore). Membranes were incubated in blocking buffer (5% skim milk/TBST [0.1% Tween, 10 mM Tris at pH 7.6, 100 mM NaCl]) for 1 hour at room temperature and then with primary antibodies diluted in 5% bovine serum albumin (BSA)/TBST overnight at 4 °C. After three washes, membranes were incubated with goat anti‐rabbit HRP‐conjugated antibody or goat anti‐mouse HRP‐conjugated antibody (7074S and 7076S, Cell Signaling Technologies; 1:5000 for both) at room temperature for 2 hours and subjected to chemiluminescence using ECL (1 856 136, Pierce).

### Luciferase Assay

For the TEAD luciferase reporter assay, 5 × 10^4^ HEK293T cells were seeded in 96‐well plates and cotransfected with pCMV‐Renilla luciferase, 8xGTIIC‐firefly luciferase, pRK5‐Myc‐TAZ, or pRK5‐FH‐NONO as indicated. After a 24‐hour incubation, firefly luciferase expression was assessed by using the Dual‐Glo Luciferase Assay System (Promega) following the manufacturer's instructions.

### Colony Formation Assay

LN229 cells were detached with trypsin, resuspended, and counted. 400 cells were seeded in each well of a 6‐well plate with complete culture medium and cultured for 10–12 days. The plates were then washed with PBS and stained with crystal violet. Colony images were scanned and scored using ImageJ.

### Tumor Sphere Assay

LN229 cells were detached with trypsin, resuspended, and counted. 500 cells were seeded in each well of a 24‐well Ultra Low Cluster Plate (Costar) with neural sphere medium (DMEM/F12, Corning #15‐090‐CV; L‐glutamine, 2mM, Invitrogen #25030‐081; N‐2 supplement, 1X, Invitrogen #17 502 048; B‐27 Supplement, 1X, Invitrogen #17 504 044; BSA, 50ug/ml, Sigma; EGF & bFGF, 20 ng/ml each, R&D systems; Antibiotic‐Antimycotic Solution, 1% Corning #30‐004‐CI) containing 0.34% low melting temperature agarose (Invitrogen #18 300 012). After solidification at 4 °C for 5 minutes, the plates were incubated at 37 °C for 2–3 weeks. Tumor spheres were stained by MTT (Invitrogen), scanned, and scored using ImageJ.

### BioID and Mass Spectrometry

To identify proteins interacting with TAZ, LN229 cells stably transduced with BirA^R118G^‐TAZ fusion protein, BirA^R118G^, or vector were seeded in 10‐cm plates containing medium with 0.5 µM Biotin for 24 h. Cells were then lysed in BioID lysis buffer (50 mM Tris HCl pH 7.4, 500 mM NaCl, 0.4% SDS, 1% Triton X‐100, 5 mM EDTA, 1 mM EGTA, and 1x protease inhibitors). After incubating with 100 µl Dynabeads MyOne Streptavidin C1 (Invitrogen #65 001) for 1 h, the precipitates were washed in washing buffer (50 mM Tris HCl pH 7.4 and 2% SDS) 3 times. The biotinylated proteins were then dissociated in 25 µl SDS sample buffer with 25 mM biotin at 95 °C. The eluted products were subjected to SDS polyacrylamide gel electrophoresis, followed by staining with GelCode Blue Stain Reagent (Thermo Scientific). Protein in each gel lane was excised from the gel. In each gel slice, protein bands were subjected to reduction with 10 mM dithiothreitol for 30 minutes at 60 °C, alkylation with 20 mM iodoacetamide for 45 minutes at room temperature in the dark, and digestion with trypsin (sequencing grade, Thermo Scientific, Cat# 90 058) overnight at 37 °C. Peptides were extracted twice with 5% formic acid and 60% acetonitrile and dried under vacuum. Samples were analyzed by nano‐liquid chromatography coupled to tandem mass spectrometry (nano LC‐MS/MS) using a Q‐Exactive HF mass spectrometer interfaced with an Ultimate 3000 RSLCnano chromatography system (Thermo Scientific). Samples were loaded on to a fused silica trap column (Acclaim PepMap 100, 75 µm x 2 cm, Thermo Scientific). After washing for 5 minutes at 5 µl/minute with 0.1% TFA, the trap column was brought in‐line with an analytical column (Nanoease MZ peptide BEH C18, 130A, 1.7 um, 75 um x 250 mm, Waters) for LC‐MS/MS. Peptides were fractionated at 300 nl/minute using a segmented linear gradient 4–15% B in 30 minutes (A: 0.2% formic acid; B: 0.16% formic acid, 80% acetonitrile), 15–25% B in 40 minutes, 25–50% B in 44 minutes, and 50–90% B in 11 minutes. Mass spectrometry data were acquired using a data‐dependent acquisition procedure with an MS1 scan (resolution 120000) followed by MS/MS (resolution 30000; HCD relative collision energy 27%) on the 20 most intense ions with a dynamic exclusion duration of 20 seconds. Considering that BirA^R118G^‐TAZ and BirA^R118G^ express at different levels as indicated by examining the input (Figure S1A, Supporting Information), counts of each protein from the mass spectrometry data in BirA^R118G^‐TAZ or BirA^R118G^‐transduced cells were normalized by the expression level of BirA^R118G^‐TAZ or BirA^R118G^, respectively, in the input. The enrichment score of each protein was then calculated as the ratio of normalized protein count from BirA^R118G^‐TAZ‐transduced cells to that from BirA^R118G^‐transduced cells. The top 50 enriched proteins based on the enrichment score are shown in Supplementary table 1.

### Immunofluorescent Staining and Chromogenic Immunodetection

Immunofluorescent staining was performed as follows ^39^. For confocal imaging to visualize EGFP‐TAZ LLPS, LN229 or HEK293T cells were seeded on glass coverslips overnight, and transfected with EGFP‐TAZ and NONO‐mCherry or myc‐Tead1 for 16 hrs. The cells were then fixed with 4% paraformaldehyde in PBS for 20 min at 4 °C and permeabilized in a PBS buffer containing 0.3% sodium deoxycholate and 0.3% Triton X‐100 for 30 min at 4 °C. After staining the nuclei briefly with DAPI, the cells were mounted in ProLong Gold Mountant (Invitrogen #P10144) and imaged imaged at a Leica SP8 inverted confocal laser scanning microscope at Penn State College of Medicine's Light Microscopy Core. When immunofluorescence staining is needed, the above permeabilized cells were blocked with 5% BSA in PBS at 4 °C for 1 hr and then incubated with indicated primary antibodies overnight at 4 °C. After three washes, cells were incubated with secondary antibodies for 2 hrs at 4 °C followed by another three washes before the DAPI staining. For histological samples, paraffin‐embedded 5‐µm sections were deparaffinized and rehydrated in successive baths of xylene and ethanol (100%, 95%, 70%, and 50%) followed by heat‐induced (95 °C) epitope retrieval in 10 mM sodium citrate buffer (pH = 6.0). After one‐hour block with 5% BSA/PBS at room temperature, samples were incubated overnight at 4 °C with primary antibodies diluted in 2.5% BSA/0.05% Triton X‐100/PBS. The next day, sections were washed three times with 0.1% Triton X100/PBS prior to incubation with secondary antibody diluted in 2.5% BSA/0.05% Triton X‐100/PBS for 60–90 minutes at room temperature. Then, sections were again washed three times with 0.1% Triton X‐100/PBS, labeled with 4,6‐diamidino‐2‐phenylindole (DAPI) for nuclear visualization, rinsed with PBS, and mounted in ProLong Gold Antifade Mountant (P10144, Invitrogen). The PLA (proximity ligation assay) was performed using the Duolink II Red Starter Kit (Sigma). Briefly, cells were prepared as above for immunofluorescence staining until being incubated overnight at 4 °C with primary antibodies. Cells were then incubated for 1 h at 37 °C with a mixture of the MINUS and PLUS PLA probes. Hybridized probes were ligated using the Ligation‐Ligase solution for 30 minutes at 37 °C and then amplified utilizing the Amplification‐Polymerase solution for 100 minutes at 37 °C. Cells were finally mounted using Duolink II Mounting Medium containing DAPI. Chromogenic immunodetection on histological samples was conducted using an Anti‐Mouse HRP‐DAB Cell & Tissue staining kit following the manufacturer's instructions (CTS002, R&D) after deparaffinization and blocking as above. All primary antibodies were diluted at a 1‐to‐100 concentration and all secondaries at 1‐to‐200 concentration unless otherwise specified.

### Antibodies, Reagents, Compounds

*Antibodies for immunohistochemistry*: NONO (sc‐166702, Santa Cruz Biotechnology) was used at a 1/200 dilution and secondary antibodies at a 1/200 dilution. *Antibodies for immunoblotting*: µ‐Actin (#3700, dilution at 1/20000), TAZ (#4883S, dilution at 1/1000), Pan‐Tead (#13 295, dilution at 1/2000), Rpb1 (#2629, dilution at 1/3000), p‐YAP^S127^ (#13 008, dilution at 1/1000), YAP (#12 395, dilution at 1/2000), p‐TAZ^S89^ (#59 971, dilution at 1/1000), Myc (#2272, dilution at 1/3000), p‐Lats1^T1079^ (#8654, dilution at 1/500), and Lats1 (#3477, dilution at 1/1000), all from Cell Signaling Technologies; HA (MMS‐101P, Biolegend, dilution at 1/3000); and goat anti‐rabbit HRP‐conjugated antibody (#7074S, dilution at 1/5000) and goat anti‐mouse HRP‐conjugated antibody, (#7076S, dilution at 1/5000) both from Cell Signaling Technologies.

### Gene Expression and Silencing

pBabe‐neo‐*TAZ* (4SA) ^32^ was generously provided by Dr. Kun‐Liang Guan. MGC Human *NONO* sequence‐verified cDNA (MHS6278‐202758488) was purchased from Dharmacon and subcloned into the pBabe‐puro vector with a FLAG‐HA‐tag on the amino‐terminus to generate pBabe‐puro‐FLAG‐HA‐*NONO*. Lentiviral vectors encoding shRNAs targeting *NONO* (#2: TRCN0000074560; #4: TRCN0000294049) were used to generate *NONO* knockdown. Lentiviral vectors encoding shRNAs targeting human *TAZ* (#07:TRCN0000370007; #69: TRCN0000019469) were used to generate *TAZ* knockdown. All shRNA‐expressing positive clones were selected via 2 µg/ml puromycin diluted in culture media. Knockdown efficiency on positive clones was verified via western blotting following selection. All shRNAs were obtained from the PSU shRNA library core facility. ON‐TARGETplus SMARTpool siRNAs against NONO, or ON‐TARGETplus siCONTROL were from Dharmacon. To knockout NONO using CRISPR, LN229 cells were transiently transfected with pSpCas9(BB)‐2A‐Puro with a guide RNA targeting the *NONO* locus. The guide sequence is GACCCAGCAGCTACTTACTC and was designed by using the Optimized CRISPR Design tool at http://crispr.mit.edu. After transfection, LN229 cells were selected with puromycin for 2 days. The transduced cells were diluted to 1 cell/well in 96‐well plates. After growing for two weeks, cell clones were expanded. NONO knockout was confirmed by western blotting.

### RNA‐Sequencing and Data Processing

RNA sequencing was performed on an Illumina HiSeq 2500 for 50 cycles using a single‐read recipe according to the manufacturer's instructions. Sequencing data were analyzed using Strand NGS. Briefly, reads were aligned to reference human genome and annotation files (GRCh38, build 38, RefSeq genes and transcripts, 2017_01_13). One‐way ANOVA was performed using a *p*‐value threshold of *p* 0.05 and fold‐change threshold of 2. For hierarchical clustering analysis, indirect relationships were chosen.

### ChIP‐Sequencing

LN229 cells were cross‐linked with 1% formaldehyde for 10 min and quenched by 0.125 M glycine solution for 5 min at room temperature. The cross‐linked cells were washed with 1 × PBS buffer for 2 times and collected. Cell pellets were resuspended in SDS lysis buffer (10 mM Tris HCl, pH 8.0, 10 mM EDTA, 1% SDS and 1X proteinase inhibitor), and then subjected to the sonication process. The sheared samples were diluted with 1X ChIP buffer (20 mM Tris HCl, pH 8.0, 0.01% SDS, 1.0% Triton X‐100, 1.0 mM EDTA, and 150 mM NaCl) and incubated with anti‐TAZ (#4883, Cell Signaling Technologies), NONO (sc‐166702, Santa Cruz Biotechnology), or rabbit IgG (#I5006, Sigma‐Aldrich) and mouse IgG(#I5381, Sigma‐Aldrich) at 4 °C overnight, followed by further incubating with protein A/G agarose beads (ThermoFisher, #20 423) for 4 h. The immunoprecipitates were subjected to a series of washing steps to remove nonspecific binding materials. After reverse cross‐linking, DNA was purified and subjected to ChIP‐sequencing. These ChIP‐DNA libraries were prepared using Illumina's TruSeq ChIP Sample Preparation Kit according to the manufacturer's instructions (Cat# IP‐202‐1012). Briefly, 10 ng ChIP DNA fragments were performed the DNA end repair using the End Repair Mix, and then purified with AMPure XP beads. After that, 3’ ends of the ChIP DNA fragments were adenylated with A‐Tailing Mix, and then ligated with adapter indices. After that, ChIP DNA fragments were amplified with the adapter primers. The quality of the DNA library was examined with Qubit and Agilent Bioanalyzer. Final libraries were submitted to paired‐end sequencing of 50 bp length on an Illumina NovaSeq 6000.

### ChIP‐Seq Data Analysis

ChIP‐seq peak calling and data analysis of transcription factor (TAZ and NONO) and histone modifications (H3K4me1, H3K4me3, and H3K27ac) ChIP‐seq reads were processed using ENCODE uniform processing ChIP‐seq pipeline (v1.4.0.1) (https://github.com/ENCODE‐DCC/chip‐seq‐pipeline2), and each sample has individual replicate using IgG ChIP‐seq as the control. Briefly, these raw data was processed through Cutadapt (version 1.2.0) program to remove adaptors and low quality reads.^[^ ^40^ ^]^ These trimmed reads were mapped to the human reference genome (hg19) using Bowtie2 with default parameters (37). The PCR duplicates reads and low mapping quality reads were filtered with Picard (http://broadinstitute.github.io/picard,v.2.22.2) and SAMtools (v1.10.0) (38). Peak calling was performed using peak calling algorithm MACS2 (v2.2.6). Genome profiles were visualized in the Integrative Genomics Viewer (IGV) browser (version 2.4.19).^[^ ^41^ ^]^ For *de novo* motif analysis, motif discovery algorithm “findmotifsgenome.pl” program was performed from the Homer (version 4.10).^[^ ^42^ ^]^ The differential peaks analysis was performed with DiffBind (v3.2.5) and DESeq2 package (Cut off: *P* value ≤ 0.05) in R language.^[^ ^43^ ^]^ The overlapping peaks analysis was carried out with “ bedtools intersect” program using Bedtools (version 2.26.0)^[^ ^44^ ^]^ and annotated by annotatePeaks.pl in Homer (version 4.10). The ChIP‐seq signals flanking the defined regions were normalized by the reads per kilobase per million mapped reads (RPKM) values, which were used to generate the heatmap plots with computeMatrix program by deepTools software (version 3.1.3).^[^ ^45^ ^]^ The visualization file of fragments or read coverages was generated by deepTools (version 3.1.3),^[^ ^45^ ^]^ including control and experimental datasets. Genome profiles were visualized in the Integrative Genomics Viewer (IGV) browser (version 2.4.19).^[^ ^41^ ^]^

### Quantitative RT‐PCR

qRT‐PCR was carried out according to standard protocols. Briefly, total RNA was extracted using TRIzol reagent (Invitrogen). cDNAs were synthesized using the iScript cDNA Synthesis kit (Bio‐Rad, 1 708 891), and qPCR was carried out on a CFX96 Touch Real‐Time PCR Detection System with SsoAdvanced Universal SYBR Green Supermix (Bio‐Rad, 1 725 271). *GAPDH* was used as an internal reference to normalize the input cDNA. Primer sequences used: *CTGF* forward, GCAGGCTAGAGAAGCAGAGC, reverse, ATGTCTTCATGCTGGTGCAG; *CYR61* forward, ACTTCATGGTCCCAGTGCTC, reverse, TGGTCTTGCTGCATTTCTTG; *GAPDH* forward, GGAGCGAGATCCCTCCAAAAT, reverse, GGCTGTTGTCATACTTCTCATGG; *CDC6* forward, TGTTCTCCTCGTGTAAAAGCC, reverse, GGGGAGTGTTGCATAGGTTGT; and *Myc* forward, GGCTCCTGGCAAAAGGTCA, reverse, CTGCGTAGTTGTGCTGATGT. The primers were designed using PrimerBank.

### Statistical Analysis

For statistical analysis (including animal studies), sample sizes were chosen based on whether differences between groups are biologically meaningful and statistically significant. No data were excluded from the analyses. For cell experiments, all cells in each experiment were from the same pool of parental cells. All mice in each experiment were from the same cohort. The mice were randomly chosen for implantation of different types of cells. For data collected by objective instruments, such as plate readers, qPCR cyclers, microscopy software, flow cytometers, animal IVIS systems, and western blotting, the investigators were not blinded to group allocation during data collection. However, investigator bias is not considered to have contributed to the data. For animal studies, the investigators were not blinded to group allocation during data collection when using the above mentioned objective instruments, but they were blinded during data analyses. Additionally, for all animal studies, randomization occurred in a blinded fashion. Statistical significance was determined as indicated in the figure legends. All center values shown are mean values, and all error bars represent standard errors of the mean (s.e.m). All statistical calculations and plotting were performed using GraphPad Prism 8. For all in vivo experiments, each data point represents an animal. For all in vitro experiments, each data point represents an average of technical replicates obtained from an independent experiment. The reproducibility of experiments (denoted by *n*) in each main figure is detailed in the corresponding legend and is summarized as number of independent experiments out of number of similar results as follows.

## Conflict of Interest

The authors declare no conflict of interest.

## Author Contributions

Y.W. and W.L. conceived the project. Y.W. performed most of the in vitro experiments with assistance from P.P.Y., Z.L., L.Z., B.A., M.T., and W.L. Animal experiments were performed by Y.W., P.P.Y., and W.L. H.L, L. Z., S.H., and W.L. performed gene expression and ChIP‐seq analysis. H.Z. performed mass spectrometry analysis. Y.W. and W.L. wrote an original manuscript. All authors provided intellectual inputs and edited the manuscript. W.L. supervised the project.

## Supporting information

Supporting Information

## Data Availability

The data that support the findings of this study will be openly available in the Gene Expression Omnibus.
