# Supplementary material for: Paraspeckle Protein NONO Promotes TAZ Phase Separation in the Nucleus to Drive the Oncogenic Transcriptional Program
Source: Adv Sci (Weinh). 2021 Oct 29;8(24):2102653. doi: 10.1002/advs.202102653 (PMC8693076; doi:10.1002/advs.202102653)
Supplement: Supplementary file 1 — Supporting Information [file ADVS-8-2102653-s001.pdf]

© 2021 Wiley-VCH GmbH

Supporting Information

for *Adv. Sci.*, DOI: 10.1002/advs.202102653

Paraspeckle protein NONO promotes TAZ phase separation in the nucleus to drive the oncogenic transcriptional program

Yiju Wei[1,], Huacheng Luo[1, 2], Patricia P. Yee[1], Lijun Zhang[3,4], Zhijun Liu[1], Haiyan Zheng[5], Lei Zhang[1,6] , Benjamin Anderson[1], Miaolu Tang[1], Suming Huang[1, 2, 7], and Wei Li[1, 4, 7 *]

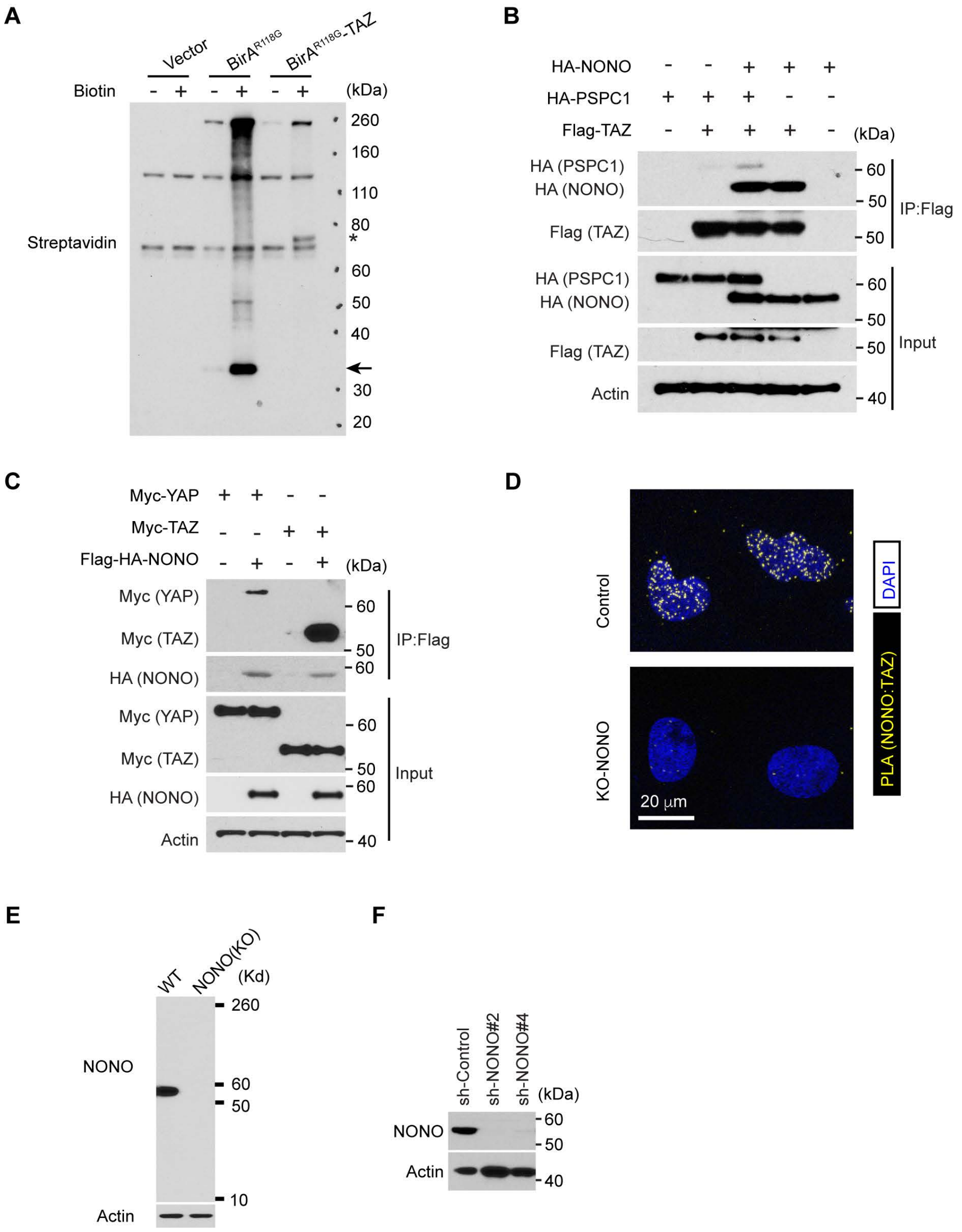

Figure S1.

(A) LN229 cells stably transduced with BirA^{R118G}-TAZ, BirA^{R118G}, or vector were cultured in medium containing 0.5 μM biotin for 24 hours. Cells were then lysed and subjected to western blotting as indicated. Arrow and asterisk indicate BirA^{R118G} and BirA^{R118G}-TAZ, respectively.

(B) and (C) HEK293T cells were transfected with indicated genes. The cells were lysed and subjected to immunoprecipitation by a Flag antibody. The immunoprecipitated products were subjected to western blotting.

(D) LN229 cells in which NONO was or was not knocked out (KO) were subjected to PLA using TAZ and NONO antibodies.

(E) LN229 cells in which NONO was or was not knocked out (KO) were subjected to western blotting.

(F) LN229 cells stably transduced with indicated shRNAs targeting NONO or a scrambled shRNA control were subjected to western blotting.

A

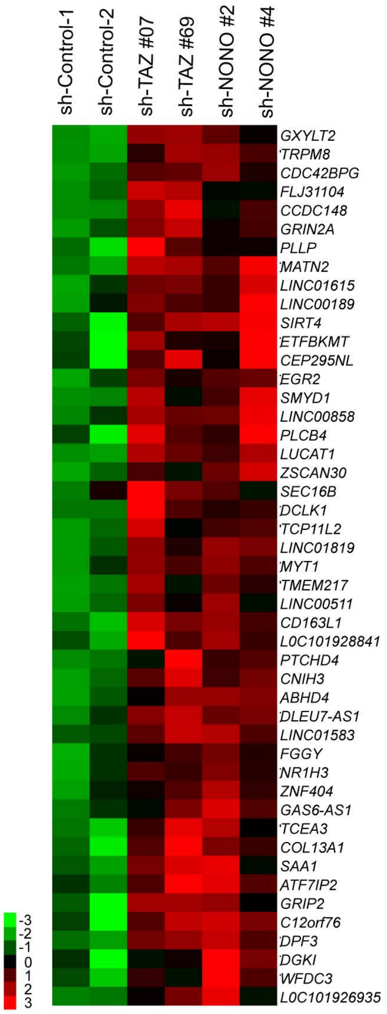

Figure S4.

(A) Genes, whose expression increases when either TAZ or NONO was knocked down by indicated shRNAs, were surveyed for TAZ and NONO peaks at their transcription promoters. The genes shown here are those having TAZ and NONO peaks at these regions. Expression of these genes (assessed by RNA-seq) was shown. The scale shows log2 fold change.

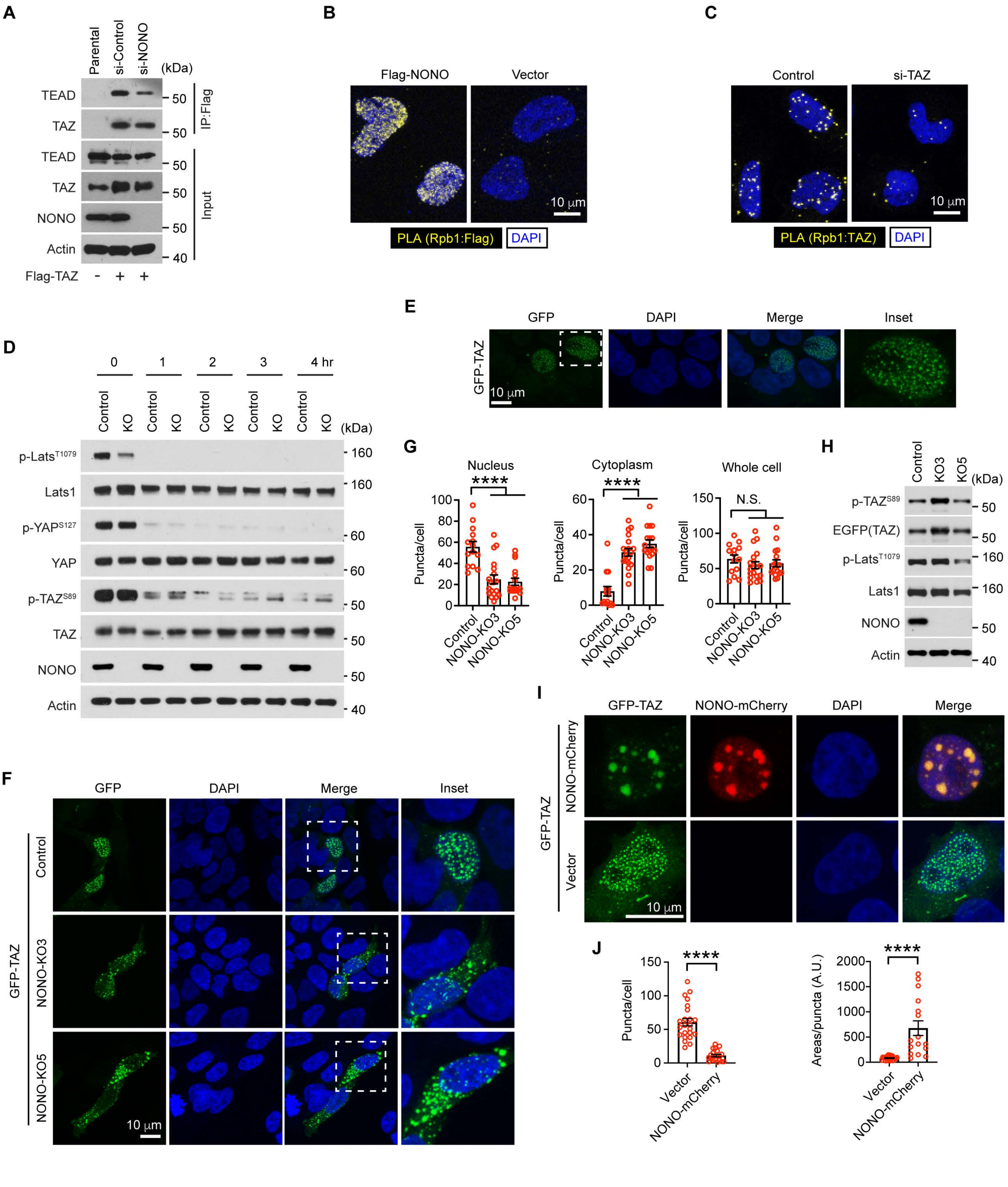
A
Parental
si-Control
si-NONO
(kDa)
TEAD
TAZ
TEAD
TAZ
NONO
Actin
IP:Flag
Input
50
50
50
50
50
40
Flag-TAZ
-
+
+
B
Flag-NONO
Vector
10 μm
PLA (Rpb1:Flag)
DAPI
C
Control
si-TAZ
10 μm
PLA (Rpb1:TAZ)
DAPI
D
0
1
2
3
4 hr
Control
KO
(kDa)
p-Lats^{T1079}
Lats1
p-YAP^{S127}
YAP
p-TAZ^{S89}
TAZ
NONO
Actin
160
160
60
60
50
50
50
40
E
GFP
DAPI
Merge
Inset
GFP-TAZ
10 μm
F
GFP
DAPI
Merge
Inset
GFP-TAZ
Control
NONO-KO3
NONO-KO5
10 μm
G
Nucleus
Cytoplasm
Whole cell
****
****
N.S.
Puncta/cell
Control
NONO-KO3
NONO-KO5
H
Control
KO3
KO5
(kDa)
p-TAZ^{S89}
EGFP(TAZ)
p-Lats^{T1079}
Lats1
NONO
Actin
50
50
160
160
50
40
I
GFP-TAZ
NONO-mCherry
DAPI
Merge
NONO-mCherry
Vector
10 μm
J
****
****
Puncta/cell
Areas/puncta (A.U.)
Vector
NONO-mCherry

Figure S5.

(A) LN229 cells stably transduced by Flag-tagged TAZ were transfected with a pool of four siRNAs against NONO or a scrambled siRNA control. These cells were then seeded on Petri dishes for 2 hours and subjected to immunoprecipitation by a Flag antibody. The immunoprecipitated products and total lysates were subjected to western blotting.

(B) LN229 cells stably transduced with Flag-tagged NONO or vector were seeded on coverslips for 4 hours and subjected to PLA using Flag and Rpb1 antibodies.

(C) LN229 cells transduced with a pool of four siRNAs against TAZ or a scrambled siRNA control were seeded on coverslips for 4 hours and subjected to PLA using TAZ and Rpb1 antibodies.

(D) LN229 cells, in which NONO was or was not knocked out (KO), were seeded (attach) on Petri dishes for indicated hours. Lysates from these cells were subjected to western blotting.

(E) HEK293T cells transiently transfected by GFP-TAZ were fixed and subjected to fluorescence imaging by confocal microscopy. Outlined areas are enlarged and shown in the insets.

(F) Control or NONO knockout HEK293T cells were transiently transfected by GFP-TAZ. After fixation, the cells were subjected to fluorescence imaging by confocal microscopy. Outlined areas are enlarged and shown in the insets.

(G) GFP-TAZ puncta in the cell nucleus, cytoplasm, or whole cell of each cell from the experiment described in (F) were quantified. Ordinary one-way ANOVA. ****P < 0.0001. N.S., P > 0.05. Each data point represents a cell with certain levels of GFP signal. $n_{control}$ = 14, $n_{NONO\text{-}KO3}$ = 19, $n_{NONO\text{-}KO5}$ = 17 cells in each condition. All cell images were collected from three independent experiments.

(H) Lysates of HEK293T cells as described in (F) were subjected to western blotting as indicated.

(I) GFP-TAZ was co-transfected with NONO-mCherry or vector into HEK293T cells. The cells were then fixed and subjected to fluorescence imaging by confocal microscopy.

(J) GFP-TAZ puncta number or size in each cell from the experiment described in (I) were quantified. Unpaired student T-test. ****P < 0.0001. Each data point represents a cell with certain levels of GFP signal. n_{vector} = 18, $n_{Nono\text{-}mCherry}$ = 11 cells in each condition. All cell images were collected from three independent experiments.

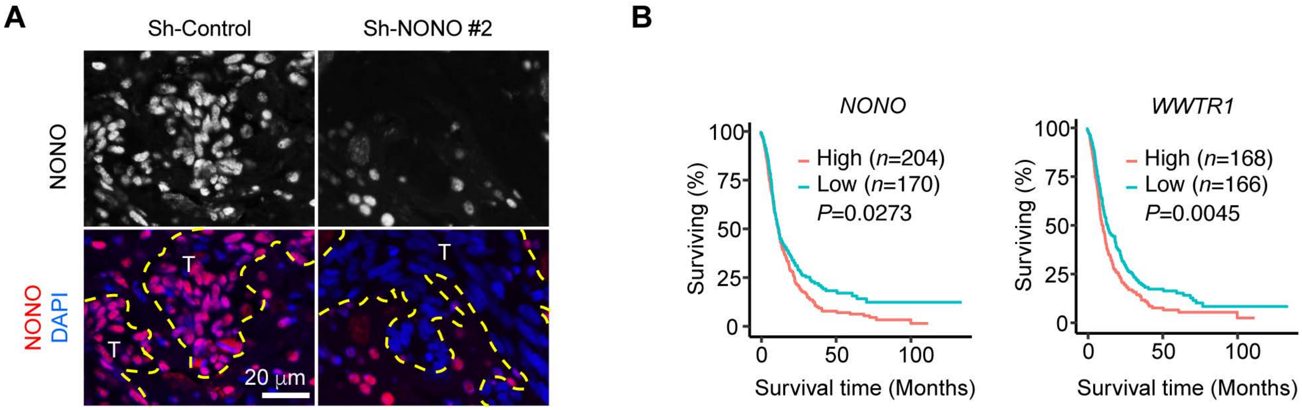

Figure S6

(A) Formaldehyde-fixed paraffin-embedded tumors derived from LN229 cells transduced with a scrambled shRNA control or shRNA targeting NONO were subjected to immunofluorescence staining for NONO. Tumor areas are outlined and indicated as T. Scale bar = 20 µm.

(B) Kaplan-Meier curves of patients showing higher or lower indicated mRNA expression. Analysis was through GiloVis using the CGGA dataset including GBM tumor types, cutoff: 6.87 (NONO) and median (WWTR1). Log-rank test. *n* indicates number of human subjects.

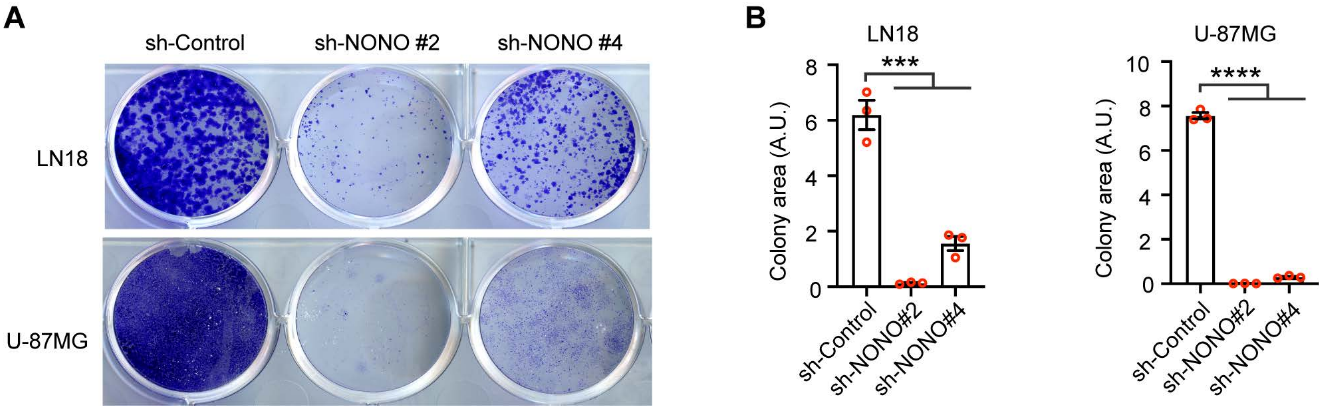

Figure S7

(A) LN18 or U-87MG cells stably transduced with indicated shRNAs targeting NONO or a scrambled shRNA control were subjected to the colony formation assay.

(B) Colony numbers in each well from the results shown in (A) were counted. n = 3, ordinary one-way ANOVA, ***P < 0.001, ****P < 0.0001.
